# Convalescent COVID-19 Patients Without Comorbidities Display Similar Immunophenotypes Over Time Despite Divergent Disease Severities

**DOI:** 10.3389/fimmu.2021.601080

**Published:** 2021-08-19

**Authors:** Chang-Feng Chu, Florian Sabath, Silvia Fibi-Smetana, Shan Sun, Rupert Öllinger, Elfriede Noeßner, Ying-Yin Chao, Linus Rinke, Elena Winheim, Roland Rad, Anne B. Krug, Leila Taher, Christina E. Zielinski

**Affiliations:** ^1^Institute of Virology, Technical University of Munich, Munich, Germany; ^2^Central Institute for Translational Cancer Research (TranslaTUM), Technical University of Munich, Munich, Germany; ^3^Infection Immunology, Leibniz Institute for Natural Product Research and Infection Biology, Hans-Knöll-Institute, Friedrich Schiller University, Jena, Germany; ^4^Institute of Biomedical Informatics, Graz University of Technology, Graz, Austria; ^5^Immunoanalytics-Tissue Control of Immunocytes, Helmholtz Zentrum München, German Research Center for Environmental Health, Munich, Germany; ^6^Institute for Immunology, Biomedical Center, Faculty of Medicine, Ludwig-Maximilians-University Munich, Planegg-Martinsried, Germany

**Keywords:** COVID-19, T cells, SARS-CoV-2, immunomonitoring, disease severity assessment

## Abstract

COVID-19, the disease caused by SARS-CoV-2 infection, can assume a highly variable disease course, ranging from asymptomatic infection, which constitutes the majority of cases, to severe respiratory failure. This implies a diverse host immune response to SARS-CoV-2. However, the immunological underpinnings underlying these divergent disease courses remain elusive. We therefore set out to longitudinally characterize immune signatures of convalescent COVID-19 patients stratified according to their disease severity. Our unique convalescent COVID-19 cohort consists of 74 patients not confounded by comorbidities. This is the first study of which we are aware that excludes immune abrogations associated with non-SARS-CoV-2 related risk factors of disease severity. Patients were followed up and analyzed longitudinally (2, 4 and 6 weeks after infection) by high-dimensional flow cytometric profiling of peripheral blood mononuclear cells (PBMCs), in-depth serum analytics, and transcriptomics. Immune phenotypes were correlated to disease severity. Convalescence was overall associated with uniform immune signatures, but distinct immune signatures for mildly *versus* severely affected patients were detectable within a 2-week time window after infection.

## Introduction

The pandemic caused by the novel SARS-CoV-2 is a global health emergency. As of now (April 2021), more than 134 million people are clinically affected by this virus, with an estimated 3-4% case-fatality rate with large regional variations reaching up to 10% (WHO status report) (1). Countries all over the world have been installing regulatory measures at unprecedented speed to prevent human-to-human viral spread. SARS-CoV-2 infection causes the disease COVID-19, which is characterized by severe respiratory symptoms. Most patients infected with SARS-CoV-2 present with mild to moderate symptoms such as fever, fatigue, cough, and anosmia (2). A small number of patients (17%), however, progress to a more severe disease course characterized by acute respiratory distress syndrome, which can ultimately lead to multiple organ failure and death (2). Comorbidities, such as cardiovascular disease, diabetes, hypertension, old age, and immunosuppressive therapies have been shown to predispose to severe COVID-19 by epidemiological investigations in a large proportion of patients (2, 3). Intriguingly, variable severity in the disease course has also been reported even in young and healthy patients (4–8). In asymptomatic infected patients, the immune system is fully capable of preventing severe disease for reasons still poorly understood. Despite this advantageous setting, this situation represents a danger to our society since the activity radius of these unaffected, though infected, individuals is high and could accelerate viral spread. It remains enigmatic what factors predispose an individual to mild *versus* severe disease in the absence of comorbidities. Despite extensive SARS-CoV2 related research efforts, clinical studies and immunomonitoring of COVID-19 patients are strongly confounded by not controlling for the presence of comorbidities and their associated medications, which are obvious and well-established risk factors for a severe disease course.

The pathogenesis of COVID-19 is strongly associated with a profound impact of host immune responses to the virus. Severe respiratory distress syndrome is associated with a cytokine storm (9, 10). Proinflammatory cytokines such as IL-2, TNF-α GM-CSF and IL-6, IL-1β, IL-8, IL-10, G-CSF have been detected in the plasma of patients with severe illness (11–13). Cytokine release is less pronounced in settings of mild disease courses, indicating a correlation with clinical features rather than infection with the virus itself (14). The innate immune compartment is rapidly activated resulting in cytokine release and tissue damage. This has initiated clinical trials with specific innate cytokine blocking drugs, such as tocilizumab (IL-6R blocking antibody) (15). They demonstrated improvement in respiratory and laboratory parameters and reduction in all-cause mortality (16–18). ACE2, the entry receptor of SARS-CoV-2, has been reported to be expressed in several innate immune cell types such as macrophages (19–21). Despite the cross-talk of innate and adaptive immunity, little is known about the differential impact of T cell abrogations on a mild *versus* severe COVID-19 disease course. Contradicting findings have been published following analysis of diverse patient groups with a range of comorbidities (22). Further analyses revealed heterogeneous roles for T cells ranging from overactivation to exhaustion in the pathogenesis within patient subgroups (23). This unmet medical dilemma needs to be disentangled in well controlled studies to identify the culprits of COVID-19 pathogenesis and thus potential therapeutic targets.

T cells participate in viral clearance through antigen specific cytotoxicity and B cell help (24, 25). Their polyfunctional cytokine release contributes to an overall immune activation with downstream inflammatory actions on immune and stromal cells (26, 27). An individual’s T cell signature could either predispose or protect from infection or severe COVID-19 illness. The T cell post-activation fingerprint represents an immune signature of protection and is expected to correspond to future vaccination success. Dissecting human T cell responses to SARS-CoV-2 will therefore yield predictive markers for the disease course as well as information on the effect of the viral infection on adaptive immunity. Recently, it has been shown that lymphopenia in both the CD8^+^ and CD4^+^ T cell compartment correlates with severe disease (14, 28, 29). T cells displayed increased activation as judged by HLA-DR upregulation or frequency of Ki67^+^ T cells (14, 30, 31). T cells reactive to spike protein in COVID-19 patients, but not in reactive healthy donors, coexpressed HLADR and CD38, which are typical for effector T cells during acute viral infection (32). However, the upregulation of inhibitory receptors such as NKG2A, PD-1 and TIM-3 hav also been reported in settings of severe illness, indicating terminal differentiation or exhaustion in response to SARS-Cov2 infection in settings of severe illness (30, 33–37). Cytokine expression in T cells from COVID-19 patients showed variable results. The percentage of IFN-γ producing CD4^+^ T cells was increased in severely affected COVID-19 patients, in line with the previously reported contribution of IFN-γ to the cytokine storm in SARS patients (38). Although IFN-γ appears to be the dominant cytokine produced by CD4^+^ T cells in COVID-19 patients, some studies reported IFN-γ to be unaltered or even decreased reflecting reduced functional T cell diversity (33, 39–42). Other T cell related cytokines showed increased levels in the serum (34, 39). These results imply that productive T cell effector function might precede a state of T cell exhaustion.

In this study, we set out to identify immune signatures of mild *versus* more severe COVID-19 in a well-controlled large patient cohort that is not confounded by comorbidities (43). We therefore implemented a high-dimensional analytical approach to characterize the composition of peripheral blood mononuclear cells longitudinally with a focus on T cell differentiation states in convalescent COVID-19 patients stratified according to the severity of their disease course. We also investigated 22 inflammatory mediators in the serum and investigated gene expression differences between severely and mildly afflicted COVID-19 patients using RNA-seq. Importantly, our patient cohort excludes study participants with relevant comorbidities or treatments, who might have skewed previous studies towards a more severe disease course, towards COVID-19-independent hospitalization and immune signatures characteristic of the underlying comorbidity and its baseline medication. This also excluded application of corticosteroids. We stratified patients according to an ordinal disease severity scoring system based on 15 COVID-19-associated symptoms and their relative strength. This strategy is instrumental for the identification of immune correlates and biomarkers of protection against SARS-CoV2 and goes beyond previous studies, which have focused on the identification of common immune signatures in highly diverse patient cohorts (44).

## Materials and Methods

### Patient Characteristics

**Figure 1:**
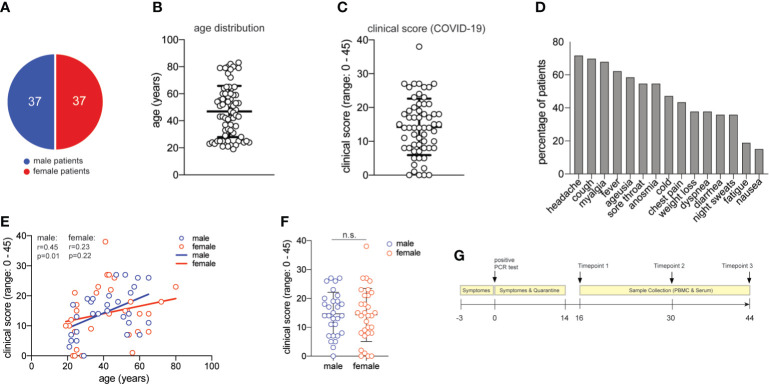
Characterization of the COVID-19 patient cohort. **(A)** distribution of male and female study participants. **(B)** distribution of the age of all study participants (mean ± SEM, 47.3 ± 2.2). **(C)** clinical score for each patient (range:0-38) as assessed by a physician assisted questionnaire with each symptom score (range: 0-3) contributing to the final score (mean ± SEM, 14.5 ± 1.1). 60 patients provided clinical metadata. **(D)** frequency of patients presenting with the symptoms assessed in the COVID-19 questionnaire. **(E)** Spearman correlation r between age and clinical score for male and female patients, respectively. **(F)** clinical scores as in **(C)** for male and female patients (unpaired student’s t test, n.s., not significant) (mean ± SEM; male: 14.7 ± 1.4; female: mean 14.3 ± 1.7). **(G)** Timeline of acquisition of patient material.

Patients were randomly selected based on positive SARS-CoV-2-RNA test results by RT-PCR from throat swab samples as well as absence of co-morbidities or pregnancy as assessed by a physician (lung diseases, cardiovascular diseases, cancer, autoimmune diseases, therapeutic immunosuppression). Patient recruitment followed a public announcement for voluntary study participation *via* the Facebook website of the Klinikum r. d. Isar, the University hospital of the Technical University of Munich. Blood sampling occurred 2 weeks after positive SARS-CoV-2-RNA test results in convalescent patients and on two additional timepoints in 2-week intervals. Patient characteristics are outlined in **Figure 1**. Clinical COVID-19 scores were assigned to each individual patient according to a questionnaire covering a wide range of 15 symptoms (**Figure 1D**), each scored 0-3 according to its degree of severity (range of clinical scores 0-45). All patients provided informed consent for this study. Ethical approval was obtained from the Ethics Review Board of the Technical University of Munich (164/20S and 147/20S) and the Ethics Review Board of the University Hospital Jena (Reg.-Nr.: 2020-1997-Material). All work involving humans was carried out in accordance with the Declaration of Helsinki for experiments involving humans.

### Cell Purification Flow Cytometry

Peripheral blood mononuclear cells (PBMC) were isolated immediately after blood collection into heparinized collection tubes (within 30 min) by density gradient centrifugation using Biocoll Separating Solution (Merck). They were analyzed immediately by flow cytometry (BDFortessa) using the following antibodies (CD45RA-BUV737, clone HI100; CD4-BV785, clone RPA-T4; CD25-BV650, clone BC96; CD8-BV510, clone RPA-T8; CD14-PacificBlue, clone HCD14; CD19-PerCP-Cy5.5, clone HIB19; CD56-FITC, clone MEM-188; CD197-PE-Cy7, clone G043H7; CD127-PE/Dazzle594, clone A019D5; CD3-PE, clone SK7; γδTCR-APC, clone B1), all from Biolegend. Dead cells were excluded by staining with Hoechst (Sigma-Aldrich). For assessment of cellular markers of acute infection such as plasmablasts, activated CD8 T cells and cTfh cells, frozen PBMC from the same patient cohort were analyzed. Cells were stained with fluorescently labeled antibodies and samples were acquired using the spectral flow cytometer Cytec Aurora (Cytek^®^ Biosciences). The antibodies are indicated in **Figure S8B** and were all purchased from Biolegend. Unmixing was performed using the software provided by the Cytec Aurora. FlowJo v10 was used for further analysis of the data.

### Cell Culture

PBMC were cultured in RPMI-1640 medium supplemented with 2 mM glutamine, 1% (vol/vol) non-essential amino acids, 1% (vol/vol) sodium pyruvate, penicillin (50 U/ml), streptomycin (50 μg/ml; all from Invitrogen) and 10% (vol/vol) fetal calf serum (Biochrom). PBMC were stimulated with plate-bound anti-CD3 (2 μg/ml, clone TR66; Enzo Life Science) and anti-CD28 (1 μg/ml CD28.2; BD Biosciences) for 3 days before intracellular cytokine staining and harvesting of supernatant.

### Intracellular Cytokine Staining

For intracellular cytokine staining, human cells were restimulated for 5 hours with PMA and ionomycin with brefeldin A added for the final 2.5 hours of culture (all Sigma-Aldrich). The cells were fixed and permeabilized with Cytofix/Cytoperm (BD Biosciences) according to the manufacturer’s instructions. The cells were stained with anti-IL-10-PerCP-Cy5.5 (clone JES3-9D7), anti-TNFa-PE-Cy7 (clone Mab11), anti-Granzyme B-FITC (clone GB11), anti-IL-17A-Pacific Blue (clone BL168), anti-IFNγ-PE-Cy7 (clone 4S.B3), anti-GMCSF-APC (clone BVD2-21C11) (all from BioLegend); and anti-IL-4-PE (clone MP4-25D2) and anti-IL-2-APC (clone MQ1-17H12) (both from BD Biosciences). Cells were analyzed with a BD LSRFortessa (BD Biosciences). Flow cytometry data were analyzed with FlowJo software (Tree Star).

### Serum Cytokine and Chemokine Analysis

Cytokines and chemokines in serum or cell supernatants were measured using the multiplex bead array system Bio-Plex human Cytokine Group I Panel 7-Plex, with added single kits for human RANTES (CCL5), human IP-10 (CXCL10), human IFN-b, IFN-a2, and IL27p28 (Bio-Rad Laboratories GmbH), following the manufacturer’s recommendations. Data were acquired using the Luminex100 machine with BioPlex Manager 6.1 software. Standard curves were fitted using the logistic-5PL regression type.

### RNA Sample and Library Preparation

Three patients with mild (m29, m42 and m43 with severity scores 7, 7 and 5) and two patients with severe (m10 and m11 with severity scores 18 and 27) disease course from the first sampled time point were chosen for RNA-seq analysis. Both groups contain patients of different gender and ages to equalize across cohorts. The two patients with a severe disease course had been hospitalized.

Samples were stained with anti-human CD14-PerCP (clone HCD14), anti-human CD45RA-FITC (clone HI100), and anti-human CD3-PE (clone SK7), all from BioLegend. Memory T Cells were defined as CD3^+^ CD45RA^–^ population and sorted with the BD FACSMelody including dead cell exclusion with Hoechst 33258. Cells were sorted directly into 96 well plates prefilled with 1X TCL lysis buffer consisting 50% 2X buffer TCL (QIAGEN), 1% 2-Mercaptoethanol (Gibco), and Nuclease-free water (QIAGEN) for RNA extraction.

Library preparation for bulk-sequencing of poly(A)-RNA was done as described previously (45). Briefly, barcoded cDNA of each sample was generated with a Maxima RT polymerase (Thermo Fisher) using oligo-dT primer containing barcodes, unique molecular identifiers (UMIs) and an adaptor. Ends of the cDNAs were extended by a template switch oligo (TSO) and full-length cDNA was amplified with primers binding to the TSO-site and the adaptor. NEB UltraII FS kit was used to fragment cDNA. After end repair and A-tailing a TruSeq adapter was ligated and 3’-end-fragments were finally amplified using primers with Illumina P5 and P7 overhangs. In comparison to Parekh et al. (45), the P5 and P7 sites were exchanged to allow sequencing of the cDNA in read1 and barcodes and UMIs in read2 to achieve a better cluster recognition. The library was sequenced on a NextSeq 500 (Illumina) with 63 cycles for the cDNA in read1 and 16 cycles for the barcodes and UMIs in read2.

### RNA-Seq Data Preprocessing and Gene Expression Quantitation

RNA-seq data was processed using the published Drop-seq pipeline (v1.0) to generate sample- and gene-wise UMI tables (46). The human reference genome (GRCh38) was used for alignment. Transcript and gene defined according to GENCODE version 34 [doi: 10.1101/gr.135350.111].

For qRT-PCR frozen PBMC were thawed and sorted for CD3^+^ (CD3-PE, clone SK7, Biolegend) and Hoechst^–^ T cells (Sigma). RNA isolation was performed with the RNeasy Mini Kit (QIAGEN). Reverse transcription was performed with the high capacity cDNA reverse transcription kit (Applied Biosystems). qRT-PCR was performed with the Taqman Gene Expression Assay probe *OASL* FAM (HS00984387_ml) and *IFI27* FAM (HS01086370_ml), 18S FAM (HS03928990_g1) (all Applied Biosystems) on the Roche Lightcycler 480 platform.

### Differential Gene Expression Analysis

Protein-coding genes were selected from the count table using the Biomart annotation for the human genome from April 2021. Differential expression analysis was performed using the DESeq2 [version 1.28.1, (47)] R (version 4.0.2, R Core Team) package. Technical replicates were combined using the collapseReplicates() function of DESeq2. Wald tests were performed using the DESeq() function. Gene expression levels were contrasted between patients with a mild COVID-19 course and patients with a severe COVID-19 course. Genes with an adjusted p-value < 0.05 and a fold-change ≥ 2 or ≤ 0.5 were considered differentially expressed.

Library size-normalized regularized log-transformed expression values were computed using the rlog() function in the DESeq2 package. We further refer to these values as “expression values”. Principal component analysis (PCA) was performed on the expression values of the DEGs using the robpca() function of the rospca [version 1.0.4, (48)] R package. Heatmaps were generated using the pheatmap R package [version 1.0.12, (49)].

Functional enrichment analysis was performed with the enrichGO() function of the clusterProfiler R package [version 3.16.1, (50)]. The annotation of the human genome in R´s org.Hs.eg.db database (version 3.11.4) was used as background. Enriched gene ontology terms were summarized using REVIGO (51) and visualized with the treemap() function of the treemap R package (version 2.4.2).

### Statistical Analyses

Error bars indicate the standard error of the mean (SEM); 2way ANOVA tests with Holm-Sidak’s multiple comparisons tests were performed to compare mildly *versus* more severely affected patients longitudinally over time*. p* values of 0.05 or less were considered significant. Analyses were performed using GraphPad Prism 9.

## Results

### Characterization of a COVID-19 Patient Cohort Without Comorbidities

Seventy-four patients were randomly selected based on the criteria of a positive SARS-CoV-2-RNA viral test result, lack of comorbidities, and balanced sex distribution (37 female, 37 male patients) (**Table S1** and **Figure 1A**). The mean age of our patient cohort was 47 years, ranging from 19 to 80 (**Figure 1B**).

We established a clinical COVID-19 severity scoring system including 15 symptoms that have been reported to occur during this disease (52). Quantitative scoring of each symptom (range 0-3) allowed to achieve a final COVID-19 severity score with a mean of 14.5 in our cohort (range: 0-45) (**Figure 1C**). More than 50% of all patients presented with unspecific symptoms such as headache, cough, myalgia, fever and a sore throat. More than 50% of all patients presented with chemosensory alterations such as ageusia and anosmia, which have been proposed to be more characteristic of SARS-CoV-2 infection and to even occur in otherwise asymptomatic infections (**Figure 1D**) (53). In our patient cohort, the chemosensory loss of taste and smell was consistently associated with other symptoms and always occurred very early in COVID-19 (*not shown*). Increased age has previously been reported to represent an independent risk factor for severe COVID-19 disease course and death (5). Accordingly, we observed slightly higher disease scores in correlation with higher age in men. Interestingly, this age-severity correlation was not significant for women (**Figure 1E**). Although men have occasionally been reported to develop more severe COVID-19 (36), severity between men and women was equally distributed in our cohort (**Figure 1F**).

We also categorized our patient cohort according to scoring systems established for clinical monitoring of COVID-19, such as the Ordinal Scale for Clinical Improvement by the WHO R&D Blueprint. Our patient cohort covered WHO scores 1-4 in the Ordinal Scale for Clinical Improvement, which ranges from 0-8. In line with many previous reports that concluded an association of very severe disease courses with comorbidities (5), our patient cohort did not require intubation and thus lacked very severe disease outcomes (**Table S1**). Our clinical symptom scale correlated significantly with the WHO ordinal scale for clinical improvement (r= 0,530; p<0,0001) (**Figure S1**).

Our patient cohort covered a range of COVID-19 severities (WHO 1-4) in the absence of comorbidities and immunosuppression, thus excluding critical predictive markers and immunological abrogations that have been previously correlated with a severe disease course. Based on these highly controlled patient characteristics, phlebotomies were scheduled approximately two weeks after the start of symptoms and two more times at 14-day intervals. At the time of blood donation, no remaining disease symptoms except for occasional fatigue were recorded in any of the patients (**Figure 1G**). In sum, we present for the first time a large COVID-19 patient cohort with a spectrum of symptoms and disease severities, which is characterized by absence of comorbidities and thus by absence of a very strong bias that might have influenced immune parameters in previous COVID-19 immune monitoring studies.

### Correlation of Immunophenotypes With COVID-19 Severity

There is substantial evidence that a dysregulated host immune response plays a critical role in disease severity in several viral infections including SARS-CoV-2 (25, 54). Comorbidities and associated therapies might predispose an individual’s immune system to a more severe disease course and even death as suggested by multiple previous studies (55, 56). However, how SARS-CoV-2 infection translates into a mild *versus* severe disease course in healthy individuals remains elusive. The host’s response to infection is mirrored by its immune signatures in the blood. We therefore set out to characterize the cellular immune cell composition in our convalescent COVID-19 patient cohort and, thereby, to correlate immunophenotypes with disease severity.

We first quantified the relative distribution of multiple immune cell subsets by multi-color flow cytometry of fresh PBMC **(**gating strategy, **Figure S2**). Convalescent COVID-19 patients were stratified into mild and a severe disease course based on their clinical severity scores (mild: ≤ 15, severe: ≥16) and assessed at three different time points after disease onset (2, 4, 6 weeks). The cut-off disease score value of 15 was chosen based on the mean disease severity score of 14.5 across all patients. We furthermore stratified our patient cohort into patients who required hospitalization or not as a surrogate for a mild *versus* more severe disease course (**Table S1**). For hospitalized patients inflammation markers such as C-reactive protein were assessed during ongoing COVID-19 two weeks prior to study enrollment (**Table S1**).

**Figure 2:**
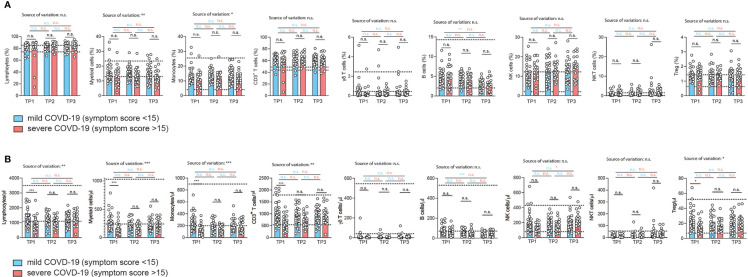
Correlation of immunophenotypes with COVID-19 severity over time. **(A, B)** Flow cytometry of *ex vivo* isolated PBMC gated on the indicated cellular subpopulations. Each circle represents one patient. **(A)** relative proportions as determined by manual gating. **(B)** absolute cell numbers as determined by manual gating. 2way ANOVA tests with Holm-Sidak’s multiple comparisons tests were performed for comparison of mildly *versus* severely affected patients longitudinally. *P < 0.05, **P < 0.01, and ***P < 0.001, n.s., not significant. The dotted lines indicate the range of physiological variations of the respective cell frequencies.

Surprisingly, the relative proportions of most immune cell types did not differ despite the divergent history of disease symptoms between the two patient groups (**Figure 2A** and **Figure S3**). However, CD14^+^ monocytes were significantly reduced two weeks after infection in the severely affected COVID-19 patients (**Figure 2A**). This alteration in the myeloid compartment was only detectable in early convalescent patients since monocyte frequencies did no longer differ from those of mildly affected COVID-19 patients upon later assessments at 4 and 6 weeks after infection. Relative frequencies of total lymphocytes and their subsets such as CD3^+^ T cells, γδ T cells, B cells, NK cells, NKT cells and Treg cells did not differ between COVID-19 patients with a mild *versus* more severe disease course at any time point analyzed (**Figure 2A**). Our longitudinal sampling of matched patient material also enabled us to conclude stability of most immune parameters across time in both severely or mildly affected patients.

To corroborate these unexpected findings, we also correlated the relative frequencies of the immune cell subsets to the respective COVID-19 symptom scores for each individual patient (**Figure S3**). We restricted our analysis to the first time point (2 weeks after infection). We did not observe any change in the relative frequency of any of the adaptive immune cell populations over the range of symptom scores in accordance with our previous analysis (**Figure S3**). The proportion of myeloid cells within the PBMCs did, however, significantly decline with increased COVID-19 severity (r=0.29, p=0.03). This was also the case for the fraction of CD14^+^ monocytes within the myeloid cell population (r=0.28, p=0.03) (**Figure S3**). We then excluded patients aged 60 and older to test if absence of this independent risk factor, as we showed in the case of men, would explain the negative correlation with myeloid cells or even unravel any new correlation with immune cell frequencies. In fact, the same results were recapitulated with a patient cohort spanning the age of 19 to 59 (mean 39 years) (data not shown). Interestingly, this demonstrates absence of any major perturbations in the relative frequencies of the main immune cell types, in particular adaptive immune cells, despite the range of COVID-19 severities in patients without relevant comorbidities. The physiological range of cell counts and frequencies is indicated by the dotted lines. Only the frequency of myeloid cells, and in particular CD14^+^ monocytes, correlated negatively with COVID-19 disease severity.

Contrary to relative immune cell proportions, the analysis of absolute numbers of immune cell subsets in PBMC revealed profound reductions in almost all immune cells in the severely affected COVID-19 patient cohort (**Figure 2B**). This showed that SARS-CoV2-induced pancytopenia across various immune cell types is masked if analysis of immune perturbations is restricted to relative frequencies of immune subsets. Only γδ T cells and B cell numbers were not affected by the disease course. This absolute loss of a wide spectrum of immune cell types in more severely affected patients was detectable in early convalescent patients (2 weeks after infection) but gradually recovered to levels indistinguishable from mildly affected patients after another 2 and 4 weeks. This suggests that even the adaptive immune compartment does not maintain major quantitative cellular alterations upon SARS-CoV2 infection. Immune signatures and biomarkers identified by multi-parameter flow cytometry are detectable in early convalescence only (**Figure 2B**).

To corroborate these conclusions, we reanalyzed all patient data using alternative disease severity definitions. This was important considering that our convalescent patient cohort, characterized by absence of relevant comorbidities, contained only patients scoring 1-4 in the ordinal scale for clinical improvement by the WHO (range: 0-8), which excludes severe disease in hospitalized patients (https://www.who.int/publications/i/item/covid-19-therapeutic-trial-synopsis) (**Table S1**). To account for the closer similarity of COVID-19 severity in our patient cohort and the overall milder disease course in the absence of cases with WHO scores greater than 5, we also stratified our patients into those with and without prior hospitalization during acute COVID-19. This showed significantly reduced frequencies of almost all analyzed immune cell subsets in patients, which had been hospitalized, at the early timepoint 1 (**Figure S4A**), which resolved at later stages. Only γδ-T cells and NK T cell frequencies did not display significant early differences in hospitalized *versus* non-hospitalized patients. Fewer significant differences in hospitalized *versus* non-hospitalized COVID-19 patients were observed upon analysis of absolute cell numbers (**Figure S4B**). Longitudinal analysis of these parameters across different timepoints within the mildly *versus* severely affected patient cohort revealed overall stability of immune parameters. However, the relative decline in lymphocytes was significantly restored over time (**Figure S4A**) in severely affected patients with stability across time in mildly affected patients. We also observed a significant increase in the percentage of CD3^+^ T cells between timepoint 1 and 3 in severly affected patients but not mildly affected patients. Also relative and absolute frequencies of NKT cells significantly increased from timepoint 1 to timepoint 3 within the severely affected patient group (**Figure S4A**).

### Correlation of COVID-19 Severity With T Cell Differentiation States

**Figure 3:**
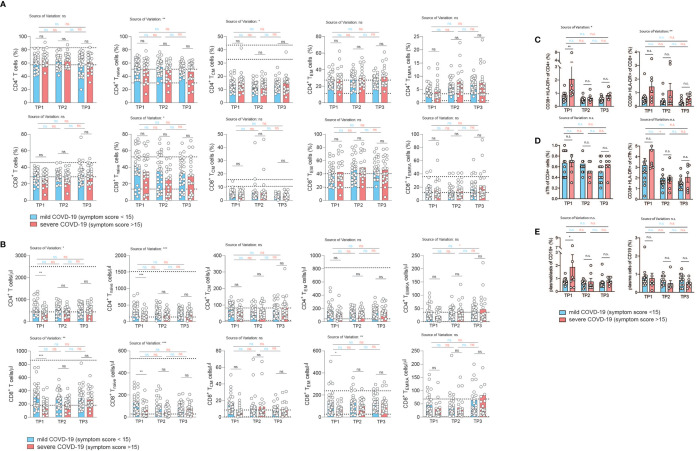
Correlation of COVID-19 severity with T cell differentiation and activation states over time. **(A, B)** Flow cytometry of *ex vivo* isolated PBMC gated on CD3^+^CD19^–^CD56^–^γδ T cell^–^ CD8^+^ or CD4^+^ T cells. T_Naive_: CCR7^+^CD45RA^+^, T_CM_: CCR7^+^CD45RA, T_EM_: CCR7^–^CD45RA^–^, T_EMRA_: CCR7^–^CD45RA^+^. Each circle indicates one patient. **(A)** relative proportions as determined by manual gating. **(B)** absolute cell numbers as determined by manual gating. **(C–E)** Spectral flow cytometry of *ex vivo* isolated PBMC (n=20). **(C)** Percentage of CD38^+^ HLA-DR^+^ activated T cells gated on CD3^+^CD4^+^ (left panel) or CD3^+^CD8^+^ T cells (right panel). **(D)** Frequency of circulating Tfh cells gated on CD3^+^CD4^+^CD45RA^–^ CXCR5^+^ PD1^+^ cells of CD4^+^ T cells (left panel), percentage of activated CD38^+^ HLA-DR^+^ cTfh cells (right panel). **(E)** Frequency of plasmablasts (gated as CD19^+^ CD27^+^ IgD^–^ CD38^+^ CD20lo CD138^–^) and plasma cells (gated as CD19^+^ CD27^+^ IgD^–^CD38^+^ CD20lo CD138^+^). Gating strategy for all cell subsets from **(C–E)** is shown in **Figure S6**. 2way ANOVA tests with Holm-Sidak’s multiple comparisons tests were performed for comparison of mildly *versus* severely affected patients longitudinally. n.s., not significant. The dotted lines indicate the range of physiological variations of the respective cell frequencies.

Memory T cells represent the repository of an individual’s antigen experience (26). A gradual loss of antigen inexperienced naïve T cells over a lifetime in accordance with a history of antigenic challenges is associated with increased frequencies of memory and effector T cells (57). The memory T cell pool contains distinct populations of central and effector memory T cells that differ in their homing capacities, functions and history of antigen encounters (25, 58). Evidence of T cell activation in both CD4^+^ and CD8^+^ T cells has recently been reported in COVID-19 patients (14, 59). We therefore decided to investigate the distribution of distinct T cell differentiation states in the CD4^+^ and CD8^+^ T cell compartment in patients following SARS-CoV-2 infection. Naïve (T_Naive_), central memory (T_CM_), effector memory (T_EM_) and terminally differentiated effector memory cells expressing CD45RA (T_EMRA_) were identified based on the differential expression of CD45RA and CCR7 within CD4^+^ and CD8^+^ CD3^+^ T cells (CD19^–^, CD56^–^, γδ TCR^–^) as described before (**Figure S2**) (58). Interestingly, we did not observe any difference between the mildly and severely affected patient cohort in the relative frequency of T cell differentiation states across the spectrum from naïve to T_EMRA_ cells at any time-point 2, 4, and 6 weeks after infection (**Figure 3A**). CD4^+^ and CD8^+^ T_Naive_ cells did, however, reveal a negative correlation across the full range of disease severity scores (CD4^+^ cells: r= -0.2974, p=0.02; CD8+ T cells: r= -0.3078, p=0.02) (**Figures S5A, B**). In CD8^+^ but not CD4^+^ T cells, this was associated with a concomitant increase in T_EM_ cells (**Figures S5A, B**). T_CM_ and T_EMRA_ cell frequencies did not correlate with disease scores. Taken together, the distribution of distinct T cell differentiation states in early convalescent COVID-19 patients was similar between the mild and severe COVID-19 patient cohort and across the range of disease severity scores, with significant changes mainly observable within T_Naive_ cells (**Figures S5A, B**).

Analysis of absolute T cell numbers, on the other hand, revealed a consistent significant reduction of naïve, T_CM_ and T_EM_ cells at the early time-point (**Figure 3B**). T_EMRA_ cells did not display any difference between the mildly and severely affected COVID-19 cohort. In the CD4^+^ T cell compartment a significant upregulation of T_EMRA_ cells was observed between the first and last timepoint within the severely affected patient cohort. Already 2 weeks later, no shifts in the absolute frequencies of T cell differentiation states were observable except for CD8^+^ T_Naive_ cells, indicating that SARS-CoV2 induced T cell alterations occur early and are not maintained on the T cell subset level (**Figure 3B**).

Reanalysis of patients who were categorized into hospitalized *versus* non-hospitalized, revealed a similar picture (**Figures S6**, **S7**). The bias for patients with a more divergent disease severity course stressed, in particular, the pronounced reduction in T_Naive_ cells in more severely affected patients across several timepoints. This was especially noteworthy in the CD8^+^ T cell compartment. Longitudinal analysis over all three timepoints showed an increase of CD8^+^ T cell frequencies between timepoint 1 and 3 within the severely affected patient group and stability among mildly diseased patients (**Figure S6B**).

We also assessed key immune populations relevant to acute infection, including activated CD4^+^ and CD8^+^ T cells, circulating T follicular helper cells (cTfh) and antibody secreting plasmablasts and plasma cells **(**gating strategy, **Figure S8**). Although T cell activation markers such as HLA-DR and CD38 showed a higher expression tendency in the more severe group, especially early after infection (TP1), the difference in the frequency of activated CD4^+^ and CD8^+^ T cells between mild and more severely affected patients was not significant (**Figure 3C****)**. There was also no difference in the frequency of cTfh cells in both patient groups (**Figure 3D**, left panel). Activation markers showed a tendency for higher expression in cTfh cells in the more severely affected COVID-19 patients at the early time point, but this was not a statistically significant difference (**Figure 3D**, right panel). This was also the case for plasmablasts (CD27^+^ CD20^lo^ CD38^+^CD138^–^, left panel) but not for plasma cells (CD27^+^ CD20^lo^ CD38^+^ CD138^+^, right panel) (**Figure 3E**).

### Cytokine Expression Profiles in T Cells From COVID-19 Patients Over Time in Correlation With Disease Severity

**Figure 4:**
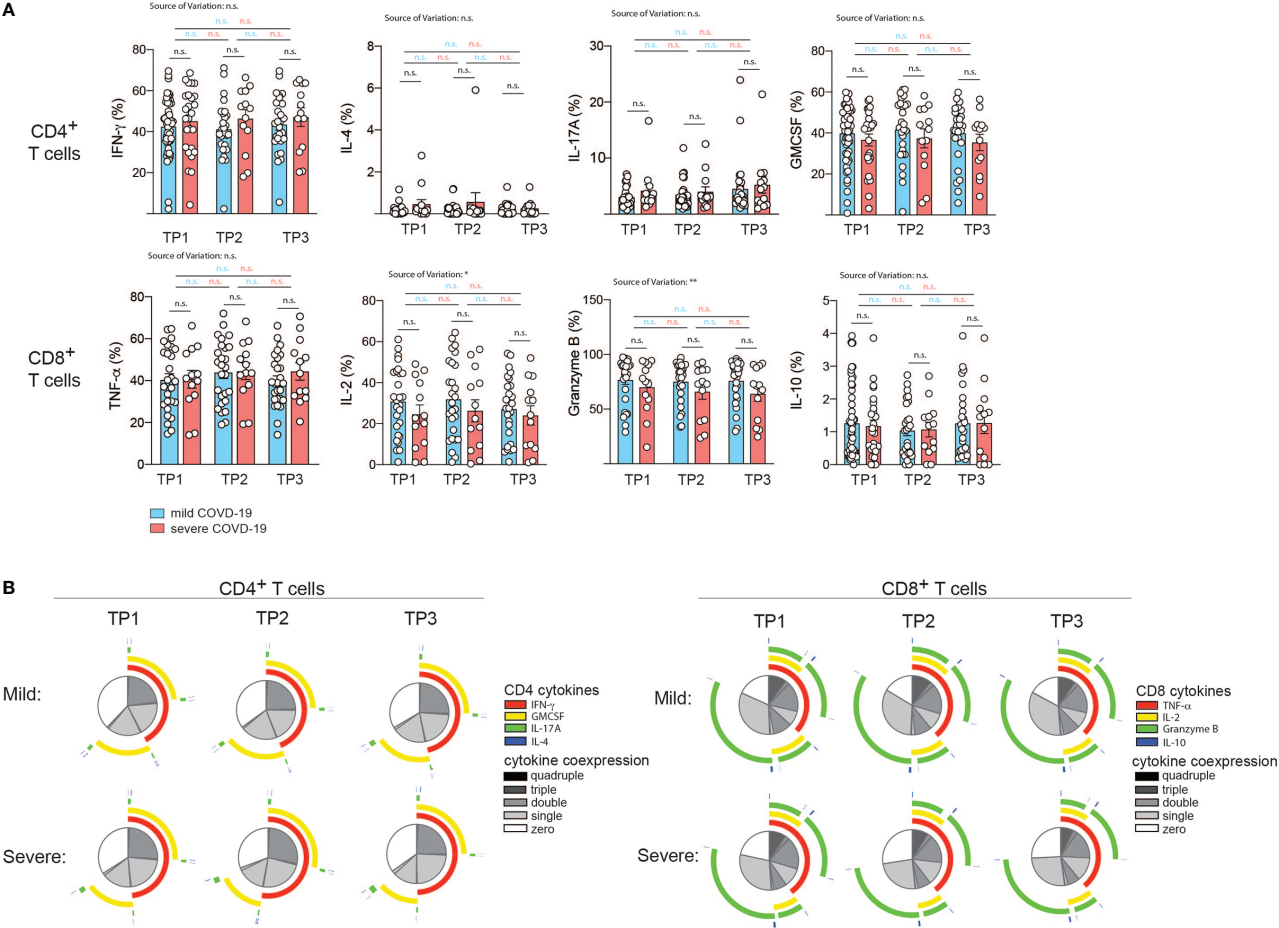
Correlation of COVID-19 severity with T cell cytokine production and T cell polyfunctionality over time. **(A, B)** Intracellular cytokine staining and flow cytometry of PBMC gated on CD3^+^ and CD4^+^ *versus* CD8^+^ T cells after 72 h of stimulation with CD3 and CD28 mAbs. **(A)**, Intracellular cytokine expression in CD4^+^ *versus* CD8^+^ T cells. Each circle represents one patient. *P < 0.05, **P < 0.01. **(B)** Polyfunctionality analysis with the SPICE software v.6 (60). The T helper cell signature cytokines IL-4, IFN-γ, IL-17A and GM-CSF were analyzed in the CD4^+^ T cell compartment. For analysis of CD8^+^ T cells, TNF-α, IL-2, granzyme B and IL-10 were analyzed.

T cell cytokine production is tailored to the specific elimination of pathogens and represents a surrogate of previous antigen exposure and the history of signal integration (25). We reasoned that SARS-CoV2 infection and consecutive perturbations of blood and tissue homeostasis in conjunction with disease manifestations of different quality and intensity would translate into changes in T cell functions. We therefore set out to longitudinally investigate the cytokine expression profiles of patients who were mildly or severely affected by COVID-19 (**Figures 4A, B**). In order to assess the functional capacity for cytokine production by T cells, we stimulated PBMC from our cohort for 72 hours with CD3 and CD28 mAbs and then performed intracellular cytokine staining of CD3 gated T cells. Interestingly, we did not observe any significant difference in the expression of the Th1, Th2, Th17 and Th_GM-CSF_, signature cytokines, which identify T helper cell subsets, i.e. IFN-γ, IL-4, IL-17A and GM-CSF respectively (26, 61, 62). This was the case for all timepoints 2, 4, and 6 weeks after infection (**Figure 4A**). No significant changes across the different timepoints were observed for mildly or severely affected patients. We also assessed the cytokine profile of CD8^+^ gated T cells. TNF-α, IL-2, granzyme B and IL-10 expression levels, which characterize cytotoxic T cell functionalities, did not differ between mildly and severely affected patients at any time-point. Expression levels were within the physiological range of interindividual variation and therefore suggested absence of any functional abrogation in T cell cytotoxicity in our patient cohort (**Figure 4A**).

The degree of cytokine co-expression has previously been reported to correlate with the efficacy of effector functions and to have an important bearing on protection (63, 64). We next assessed the polyfunctionality of CD4^+^ T helper and CD8^+^ cytotoxic T cells based on cytokine coexpression patterns. Interestingly, no significant difference in the fraction of single, double, triple or quadruple cytokines was observed at any time-point despite the divergent disease course between the two patient groups. This also applied to the type of overlapping cytokines (**Figure 4B**).

Taken together, this indicates that bulk T helper and cytotoxic T cell populations display comparable effector functions with respect to cytokine production after a mild *versus* severe COVID-19 disease course in the absence of comorbidities and do not leave an immunologic footprint indicative of the preceding disease course.

To corroborate our findings, we also assessed patients categorized according to their hospitalization status (**Figure S9**). Hospitalized patients displayed significantly upregulated IL-10 expression compared to non-hospitalized patients at early (TP1) and late (TP3) timepoints by cytotoxic T cells with a similar trend at TP2. T helper cells from hospitalized patients showed higher IL-4 expression at the late timepoint (TP3) (**Figure S9**). Longitudinal analysis across different timepoints within the hospitalized and non-hospitalized patient groups revealed stable cytokine expression levels (**Figure S9**). This indicates that patient stratification with an emphasis for highlighting the most severely affected patient group unmasks cytokine alterations. They demonstrate anti-inflammatory features in more severely affected COVID-19 patients.

### Profiling Serum Cytokines in Correlation With COVID-19 Severity Over Time

Serum cytokines have previously been profiled extensively in various diseases to identify biomarkers and predictive markers of disease progression. Increases of systemic cytokine concentrations usually indicate very strong aberrations of immunity since cytokines act as local mediators. Recent investigations in COVID-19 patients have reported elevated levels of inflammatory mediators such as IL-6, which suggests a contribution of this and other cytokines as well as their innate cell sources to the systemic cytokine response that is characteristic of the COVID-19 acute respiratory distress syndrome (ARDS) (65, 66).

**Figure 5:**
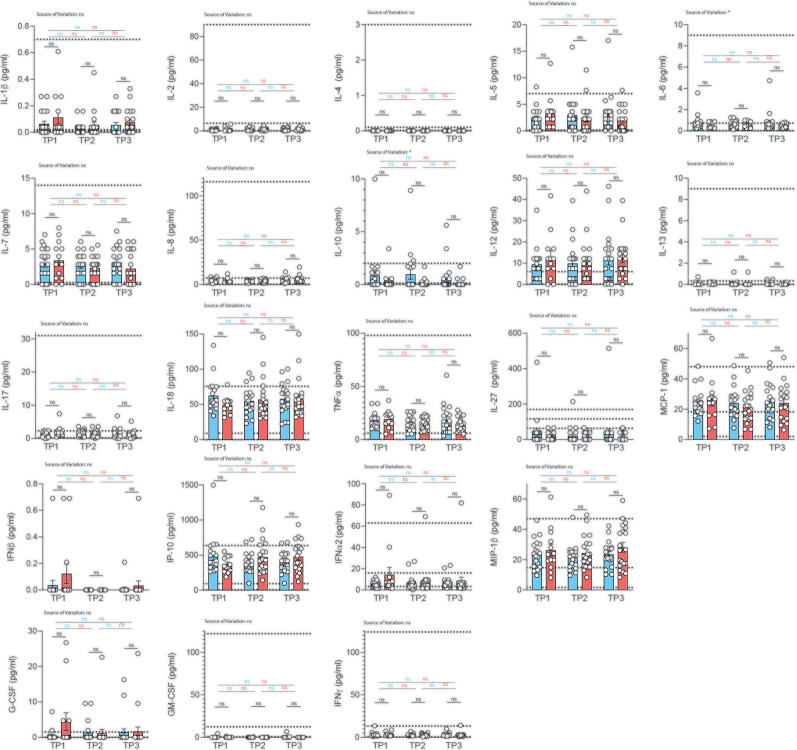
Analysis of serum cytokines over time in COVID-19 patients with different disease severity. Serum was analyzed for cytokines using the multiplex bead array system Bio-Plex. Each circle represents one patient. Horizontal dotted lines indicate the physiological range of cytokine concentrations (low and high range, mean). 2way ANOVA tests with Holm-Sidak’s multiple comparisons tests were performed for comparison of mildly *versus* severely affected patients longitudinally. *P < 0.05; n.s., not significant.

So far, most studies have been conducted in hospitalized patients, leaving systemic cytokine signatures in convalescent patients largely unexplored. Importantly, no information on serum cytokines is available to date in a COVID-19 cohort without comorbidities that could bias serum analysis. We therefore decided to profile serum cytokines in convalescent patients longitudinally to identify if and how long immune cytokine responses of a particular quality would be detectable systemically and whether they would correlate with previous disease severity (**Figure 5**).

We profiled 22 cytokines in the serum of mildly and severely affected convalescent patients at three distinct time-points (2, 4, 6 weeks after infection) and assessed serum cytokines with a multiplex bead array system (Bio-Plex). Interestingly, we did not observe any significant difference in serum cytokine concentrations between the mildly and more severely affected COVID-19 cohort at any timepoint for any of the cytokines (**Figure 5**). No significant variation was observed across different timepoints within mildly or severely affected patients. This was corroborated across disease severity scores for the early time-point (**Figure S10**). These data were unexpected considering the divergent range of disease symptoms covering asymptomatic and hospitalized patients prior to serum analysis. However, transient early changes in innate cytokine release could have potentially masked differences between the patient groups upon analysis at the convalescent stage.

Convalescence was overall associated with rather uniform immune signatures across a wide range of disease severities. This was corroborated using several different disease severity scoring strategies such as disease symptom severity scores, hospitalization status as well as the WHO Ordinal scale for Clinical Improvement (**Figures S11**, **S12**). Although significant alterations in the distribution of immune cell subsets and T cell differentiation states were observed early after disease resolution, they did not persist for longer than two weeks in most cases.

### Gene Expression Differences Between a Mild and Severe COVID-19 Course

**Figure 6:**
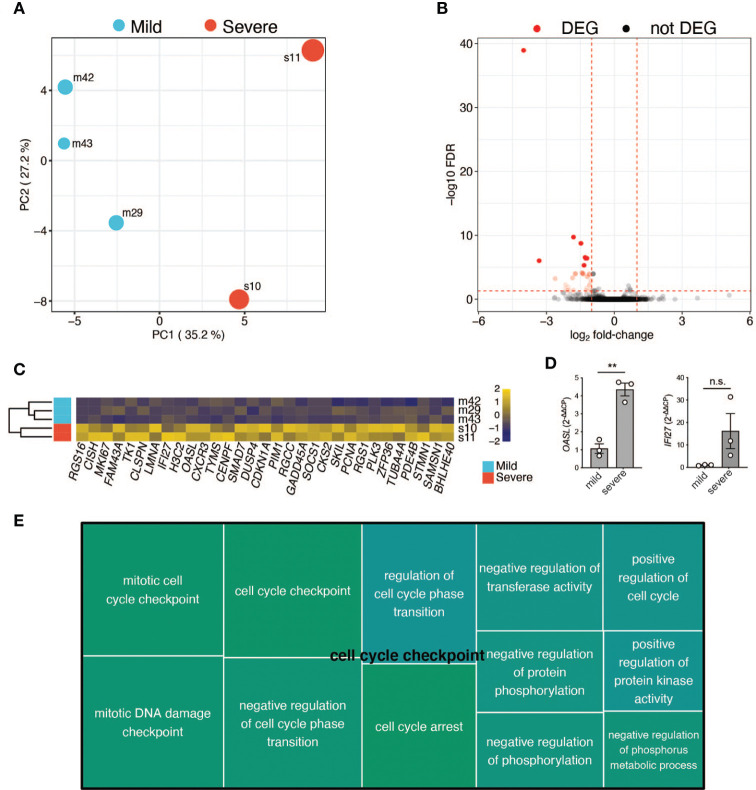
RNA-Seq analysis of selected patients. **(A)** Principal component analysis based on the expression values of 16,282 protein-coding genes. First (PC1) and second (PC2) principal components. The size of the dots represents the severity score of the corresponding sample. PC1 explains ~40% of the variance and separates patients with a mild COVID-19 course from the ones with a severe COVID-19 course. **(B)** Volcano plot showing the adjusted p-values and fold-changes in gene expression levels of patients with a mild COVID-19 course relative to patients with a severe COVID-19 course. DEGs are highlighted in red. **(C)** Heatmap showing the row-scaled regularized log transformed counts of the DEGs. Each column represents a gene and each row, a patient. The genes are ordered according to their fold-change, with the most down-regulated gene in mildly affected patients on the left. **(D)** qRT-PCR of CD3^+^ T cells from three independent patients within the mildly *versus* severely affected COVID-19 patient groups (TP1). Unpaired student’s t test. **P < 0.01; n.s., not significant. **(E)** Tree map of biological process gene ontology (GO) terms enriched among the DEGs. Only GO terms with at least 5 impacted genes are displayed. Each GO term is represented by a rectangle with a size corresponding to its p-value. Related GO terms are visualized with similar colors.

Finally, we used RNA-seq to assess global gene expression differences between the memory T-cells of three random patients with mild COVID-19 disease course and two with severe COVID-19 disease course (patient characteristics in **Tables S1****,** **S2**). We generated an average of ~4 million read pairs for each patient (see **Table S3**) and successfully mapped ~90% of them to the human genome, quantifying 16,282 (protein-coding) genes. PCA on the expression values of these genes revealed that the patients clustered according to the severity of the disease (**Figure 6A**; Materials & Methods). In particular, the first principal component (PC1) separated the patients with a mild COVID-19 course from those with a severe course and accounted for ~40% of the variance in the data. Surprisingly, we only identified 31 differentially expressed genes (DEGs) between the two patient groups (**Table S3**; Materials and Methods), with all of them (30) being downregulated in the more mildly affected patients (**Figures 6B, C**). DEGs were mainly associated with cell cycle processes such as regulation of mitotic cell cycle phase transition and regulation of cell cycle phase transition (**Figure 6E**), indicating a less vigorous expansion of memory T-cell in more mildly affected COVID-19 patients. Of note, *OASL* was significantly upregulated in severely affected COVID-19 patients (**Figure 6C**). This bioinformatic transcriptome analysis with few patient samples was further validated by targeted qRT-PCR in more patients. IFN-inducible oligoadenylate synthetases-like (OASL) protein has previously been reported to have antiviral activity by mediating RIG-I activation, thus contributing to host antiviral responses (67). Likewise, antiviral activity has previously been attributed to interferon Alpha inducible Protein 27 (IFI27) (68), which was significantly upregulated upon transcriptomic analysis (**Figure 6C**). Comparison of three further mildly *versus* severely affected patients by qRT-PCR analysis showed a tendency for IFI27 upregulation, which did, however, not reach statistical significance (**Figure 6D**). A role of these molecules as potential biomarkers for previously mildly *versus* severely affected patients at the convalescent stage needs to be investigated in more detail in the future.

## Discussion

The SARS-CoV-2 pandemic has stirred research efforts with tremendous speed and dedication all over the world. Several studies investigating innate and adaptive immunity have been initiated (69, 70). Comparisons are complicated by variable patient cohorts, which differ in age, sex, time after infection, disease course, comorbidities and medical treatment. We therefore set out to provide a unique patient cohort excluding previously identified risk factors for a severe COVID-19 disease course. Importantly, patients were all investigated at similar time-points defined by the end of quarantine, which was legally imposed by the German government for two weeks after PCR-confirmed diagnosis of SARS-CoV-2 infection. In all convalescent patients, classical parameters of inflammation such as C-reactive protein and ferritin were within the physiological range (non shown), thus prompting further investigation of multi-dimensional immune parameters that would correlate with disease severity.

Despite a wide range of disease severities, our patient cohort presented with overall very similar convalescent immunophenotypes starting approximately 4 weeks after disease onset that did not correlate with disease scores. Although we did not perform a direct comparison to healthy PBMC samples, the immunophenotypes that we obtained were within the physiological ranges reported before (71), thus excluding any major immune perturbations following SARS-CoV-2 infection. Analysis 14 days earlier did, however, reveal a remarkable transient pancytopenia in all major immune cell types. Of note, significant alterations were only maintained within the myeloid cell compartment, which demonstrated reduced cell numbers and reduced frequencies of CD14^+^ monocytes in correlation with increased disease severity. This was remarkable considering the short life of innate as compared to adaptive immune cells. SARS-CoV-2 precipitates monocyte influx and macrophage accumulation in the lung during active infection according to previous reports (72). However, sequestration of monocytes into peripheral organs, such as the lung, or activation triggered loss can hardly explain the long maintenance of their relative reductions in convalescence. Monocytes have been reported to be directly infected by SARS-CoV2. Although this occurs rather inefficiently due to the absence of ACE2 expression, infection can be increased by hypoxia and its downstream upregulation of a glycolytic metabolism by hypoxia inducible factor 1alpha (Hif1α) (73, 74). It remains elusive whether SARS-CoV2 infection can affect the bone marrow and thus the *de novo* supply of innate immune cells (75, 76). The cytopenia could have also been caused by the systemic cytokine response that is not detectable anymore at convalescence. Taken together, the data indicate overall stability of the immune composition in early convalescent patients over a wide range of disease severities, but also reveal a short time window with profound pancytopenia across multiple cell types in the very early convalescent symptom-free phase after infection.

Identifying key T cell response metrics is relevant for vaccination strategies and predictions about their durability. T cells specific for SARS-CoV2 have recently been detected within T_CM_, T_EM_ and T_EMRA_ cells, but their characteristics and role in COVID-19 pathogenesis remained undefined (77). Interestingly, we observed stronger reductions in the absolute numbers of early compared to very late differentiated T cells of the CD4^+^ and CD8^+^ lineage in mildly *versus* severely affected COVID-19 patients. While T_NAIVE_, T_CM_ and T_EM_ cells were reduced, T_EMRA_ cells did not display any difference in cell numbers between the two patient groups. This T cell loss was only evident in early convalescence but not anymore 4 weeks after infection. Moreover, a transcriptomic comparison of randomly selected severely *versus* mildly affected COVID-19 patients revealed a high degree of similarity, although a significant upregulation of the cell cycle checkpoint associated supercluster was observed two weeks after infection. Taken together, the data indicate that immune perturbations in response to SARS-CoV2 infection are only transient on a quantitative level.

The loss of T cells is poorly understood, but not likely related to direct infection of T cells with the virus due to absence of any relevant viral entry receptor (74). The strong relative reduction of T cells within the naïve T cell compartment is striking considering that that this did not translate into a concomitant increase in memory and effector T cells. This suggests loss from the blood circulation due to migration or sequestration into peripheral tissues. However, comparative analysis of bronchoalveolar lavage fluid (BAL) from moderate *versus* severely affected COVID-19 patients demonstrated lower levels of CD8^+^ T cells in BAL in patients with the most overt lymphopenia (78). Nevertheless, activated T cells with resident memory and effector memory phenotype are preferentially found in the airways compared to the circulation (79). T cell expansion in the pancytopenic microenvironment due to homeostatic cytokine signaling may have shifted naïve T cells into the memory T cell pool (virtual memory) and possibly helped reconstitute total cell numbers over time. A high proportion of cross-reactive T cells, which were potentially generated in response to common cold viruses, have recently been proposed to correlate with a milder disease course (80, 81). In fact, it has been postulated that even 20-81% of SARS-COV-2-unexposed individuals harbor T cells that respond to SARS-CoV-2 antigens, presumably due to cross-reactivity with Common Cold coronaviruses or other antigens (82). This also suggests that memory and effector T cells of previously SARS-CoV2 unexposed individuals will respond to a first-time infection with the virus. Future antigen specific T cell assays and, in particular, T cell receptor clonality analyses will help to further elucidate the dynamics of SARS-CoV-2 immunological memory formation and shifts between the respective T cell differentiation states. Interestingly, recent data demonstrated that CD8^+^ T cells with specificities against conserved peptides amongst a variety of coronaviruses are much more abundant in COVID-19 patients with a mild disease *versus* those with a more severe disease, which is suggestive for a protective role (83). This knowledge is relevant for the design of vaccines. Longitudinal investigations within the same patient cohort will be critical to assess the stability of the immune response to SARS-CoV2 infection in correlation with disease severity (84).

SARS-CoV2 infection can cause a systemic cytokine response, which has been associated with several detrimental clinical features of COVID-19, most importantly the acute respiratory distress syndrome (ARDS). In particular innate cytokines, such as IL-6, were reported to be highly elevated during acute disease and thus to represent relevant drug targets but also predictors of the disease course (85). However, a recent study suggested that inflammatory cytokines were not higher in severe COVID-19 patients than in moderate or critically ill patients with ARDS or sepsis (86). This indicates a correlation of cytokine levels with the clinical symptoms irrespective of the causative agent. Our in-depth analysis of 22 cytokines did not reveal any difference in mildly *versus* severely affected COVID-19 patients upon convalescence. This indicated a rapid restoration of systemic cytokine levels upon clinical recovery and that cytokine levels might only be good predictors of patient survival if assessed very early upon disease onset (87). The prolonged detection of elevated inflammatory cytokines, which is observable in severe ongoing COVID-19, is, however, an indicator of an adverse outcome in this group of patients (13).

Taken together, this study highlights a wide distribution of disease severities in a well characterized and standardized COVID-19 patient cohort despite absence of any relevant previously reported risk factors, in particular old age, comorbidities and therapeutic immunosuppression. Despite the spread of disease severities, the immune composition in convalescent patients quickly normalized within only 2 weeks after observable pancytopenia and shifts in T cell differentiation states. The reanalysis of our data using several disease scoring strategies supports our conclusions. The immune signatures after infection with SARS-CoV-2 represent immune correlates of viral clearance and clinical recovery and most likely also of protection from reinfection in correlation with symptom scores. High-dimensional data analyses including single-cell RNAseq and CyTOF, which are currently further elucidating the intricacies of COVID-19 associated immune signatures are expected reveal more robust insights into the cellular activation states and T cell exhaustion and might serve as cellular biomarkers (88, 89). Although most single-cell studies with clinical material suffer the caveats of limited patient numbers and patient cohort stratification, they provide in-depth insights into the transcriptional regulation of the cellular target population or allow correlations with complex cellular subgroups that are identified based on differential gene expression patterns (23, 90). Furthermore, in-depth analysis of immune correlates of infection, clinical severity and protection in peripheral tissues that are affected by COVID-19 have recently emerged and complement our and other studies, which focus on the recirculating immune system (91). IL-33 has, for example, emerged as a molecular biomarker for severe COVID-19 by BAL analysis (92). Cumulatively, these findings educate clinical trials and should inform therapeutic strategies, in particular vaccine design.

## Data Availability Statement

RNA-seq raw data presented in this study were deposited in GEO under the accession number GSE181032.

## Ethics Statement

The studies involving human participants were reviewed and approved by Ethics committee of the Klinikum r.d.Isar of the Technical University of Munich (164/20S and 147/20S) and the Ethics committee of the University of Jena (2020.1997-Material). The patients/participants provided their written informed consent to participate in this study.

## Author Contributions

C-FC, SS, Y-YC, LR, EW, and FS performed the experiments and analyzed the data. RÖ performed the RNA-seq, which was supervised by RR. EN, SF-S, AK and LT analyzed data. CZ conceived and designed the study, interpreted the data and wrote the manuscript. All authors contributed to the article and approved the submitted version.

## Funding

This work has been supported by the SFB1054 (project B10 and A06, project-ID 210592381), SFB1335 (project P18, project-ID360372040), TRR237 (project B14), Collaborative Research Centre (CRC)/Transregio124 FungiNet (project C7, project number 210879364) from the German Research Foundation (DFG) and the Carl-Zeiss-Stiftung.

## Conflict of Interest

CZ has received consulting fees from Novartis and Janssen Pharma. They are unrelated to the contents of the manuscript.

The remaining authors declare that the research was conducted in the absence of any commercial or financial relationships that could be construed as a potential conflict of interest.

## Publisher’s Note

All claims expressed in this article are solely those of the authors and do not necessarily represent those of their affiliated organizations, or those of the publisher, the editors and the reviewers. Any product that may be evaluated in this article, or claim that may be made by its manufacturer, is not guaranteed or endorsed by the publisher.

## Supplementary Material

Supplementary Figure 1 Correlation of ordinal symptom and disease scores. Correlation of clinical symptom scores covering the symptoms shown in **Figure 1D** with the WHO ordinal scale for clinical improvement (range: 0 (uninfected) – 8 (dead)). r= 0.53, p<0.0001.

Supplementary Figure 2 Gating strategy for immune cell types and T cell differentiation states. Shown is a representative gating strategy by flow cytometry for one patient.

Supplementary Figure 3 Correlation of immunophenotypes with COVID-19 disease severity scores. Flow cytometry of *ex vivo* isolated PBMC gated on the indicated subpopulations. Each circle represents one patient. *r*, Pearson correlation coefficient, *p*, p-value.

Supplementary Figure 4 Correlation of immunophenotypes with the hospitalization state over time. **(A, B)** Flow cytometry of *ex vivo* isolated PBMC gated on the indicated cellular subpopulations. Each circle represents one patient. **(A)** relative proportions as determined by manual gating. **(B)** absolute cell numbers as determined by manual gating. 2way ANOVA tests with Holm-Sidak’s multiple comparisons tests were performed for comparison of mildly *versus* severely affected patients longitudinally. n.s., not significant.

Supplementary Figure 5 Correlation of T cell differentiation states with COVID-19 disease severity scores. Flow cytometry of *ex vivo* isolated PBMC gated on the indicated subpopulations. Each circle represents one patient. *r*, Pearson correlation coefficient, *p*, p-value.

Supplementary Figure 6 Correlation COVID-19 hospitalization and disease severity with T cell differentiation states. **(A, B)** Flow cytometry of *ex vivo* isolated PBMC gated on CD3^+^CD19^–^CD56^–^γδ T cell^–^ CD8^+^ or CD4^+^ T cells in hospitalized *versus* non-hospitalized patients. T_Naive_: CCR7^+^CD45RA^+^, T_CM_: CCR7^+^CD45RA, T_EM_: CCR7^–^CD45RA^–^, T_EMRA_: CCR7^–^CD45RA^+^. Each circle indicates one patient. Shown are relative proportions as determined by manual gating. 2way ANOVA tests with Holm-Sidak’s multiple comparisons tests were performed for comparison of mildly *versus* severely affected patients longitudinally. n.s., not significant.

Supplementary Figure 7 Analysis of absolute numbers of T cell subsets in the blood in COVID-19 patients stratified into hospitalization and disease severity. **(A, B)** The analysis was performed as in S7. Shown are absolute cell numbers per μl blood. A two-tailed unpaired *t* test was performed for comparison of the respective patient groups. n.s., not significant.

Supplementary Figure 8 Gating strategy for cellular markers of acute infection. **(A)** Shown is a representative gating strategy by spectral flow cytometry for one patient (left panel). **(B)** antibodies and their respective fluorochromes (right panel).

Supplementary Figure 9 Analysis of cytokine production in COVID-19 patients stratified into hospitalization and disease severity. Intracellular cytokine staining and flow cytometry of PBMC gated on CD3^+^ and CD4^+^ *versus* CD8^+^ T cells after 72 h of stimulation with CD3 and CD28 mAbs in hospitalized *versus* non-hospitalized COVID-19 patients. Each circle represents one patient. 2way ANOVA tests with Holm-Sidak’s multiple comparisons tests were performed for comparison of mildly *versus* severely affected patients longitudinally. n.s., not significant.

Supplementary Figure 10 Correlation of serum cytokines with COVID-19 disease severity. Serum was analyzed for cytokines using the multiplex bead array system Bio-Plex. Each circle represents one patient. Horizontal dotted lines indicate the physiological range of cytokine concentrations (low and high range and mean). *r*, Pearson correlation coefficient, *p*, p-value.

Supplementary Figure 11 Analysis of the relative proportions of immune cell subsets and T cell differentiation states in convalescent COVID-19 patients stratified according to the WHO Ordinal Scale for Clinical Improvement. Flow cytometry of *ex vivo* isolated PBMC gated on the indicated cellular subpopulations. Each circle represents one patient. Shown are the relative proportions of immune cell subsets. **(A)** Shown are immune cell types. **(B)** Shown are T cell differentiation states.

Supplementary Figure 12 Analysis of the relative proportions of absolute numbers of immune cell subsets and T cells in distinct differentiation states in convalescent COVID-19 patients stratified according to the WHO Ordinal Scale for Clinical Improvement. Flow cytometry of *ex vivo* isolated PBMC gated on the indicated cellular subpopulations. Each circle represents one patient. Shown are the absolute cell numbers of immune cell subsets. **(A)** Shown are immune cell types. **(B)** Shown are T cell differentiation states.

Supplementary Table 1 Patient information.

Supplementary Table 2 Quantitative assessment of individual COVID-19 symptoms for patient characterization.

Supplementary Table 3 Differentially expressed genes for the comparison of severely *versus* mildly affected COVID-19 patients two weeks after infection.
