# Supplementary figures and images for: Convalescent COVID-19 Patients Without Comorbidities Display Similar Immunophenotypes Over Time Despite Divergent Disease Severities
Source: Front Immunol. 2021 Aug 19;12:601080. doi: 10.3389/fimmu.2021.601080 (PMC8634761; doi:10.3389/fimmu.2021.601080)

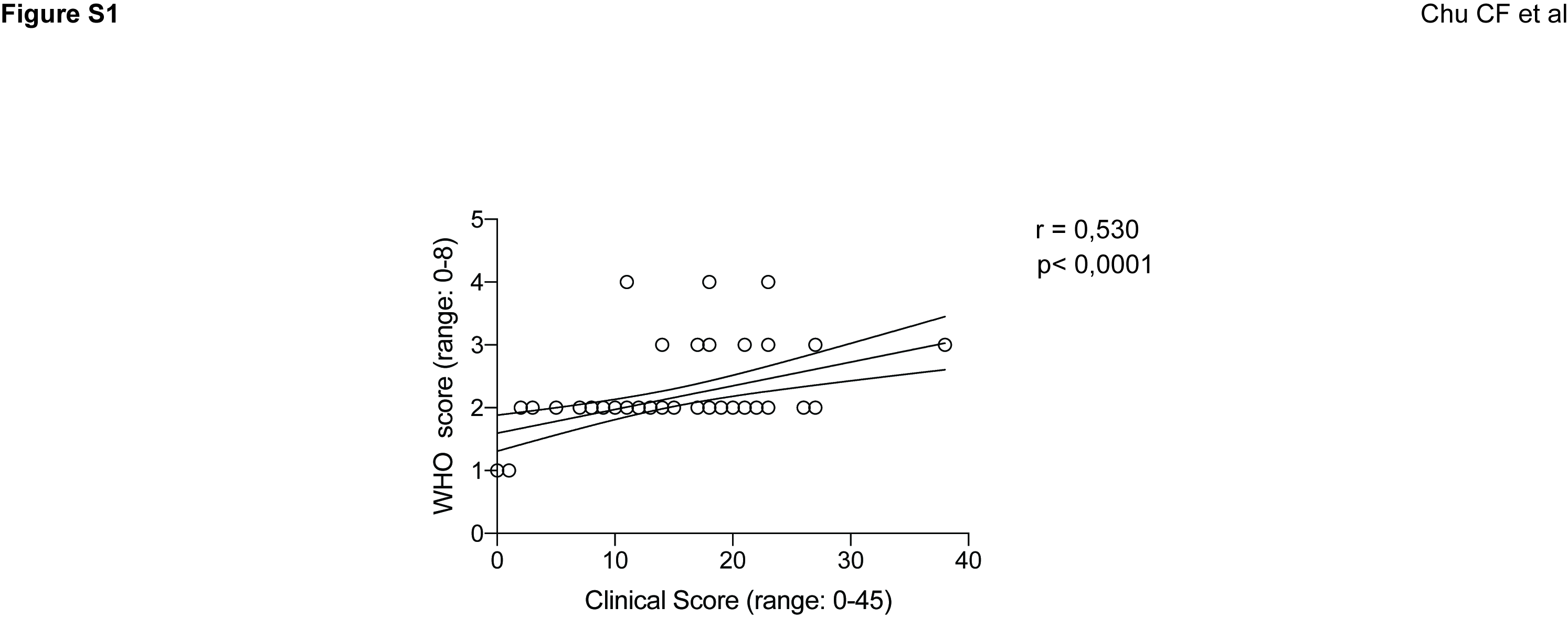

Supplement: Supplementary Figure 1 — Correlation of ordinal symptom and disease scores. Correlation of clinical symptom scores covering the symptoms shown in Figure 1D with the WHO ordinal scale for clinical improvement (range: 0 (uninfected) – 8 (dead)). r= 0.53, p<0.0001. [file Image_1.tif]

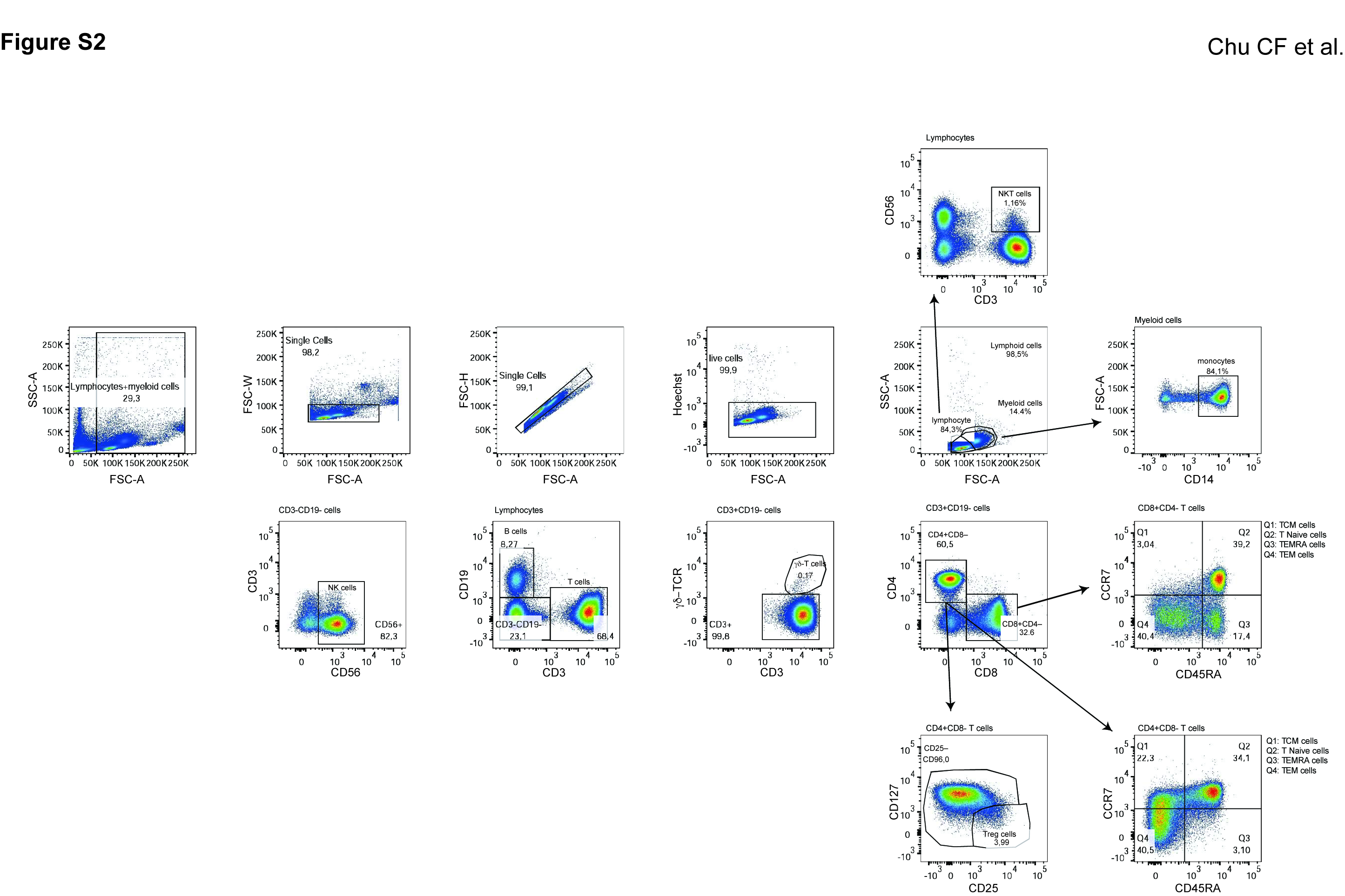

Supplement: Supplementary Figure 2 — Gating strategy for immune cell types and T cell differentiation states. Shown is a representative gating strategy by flow cytometry for one patient. [file Image_2.tif]

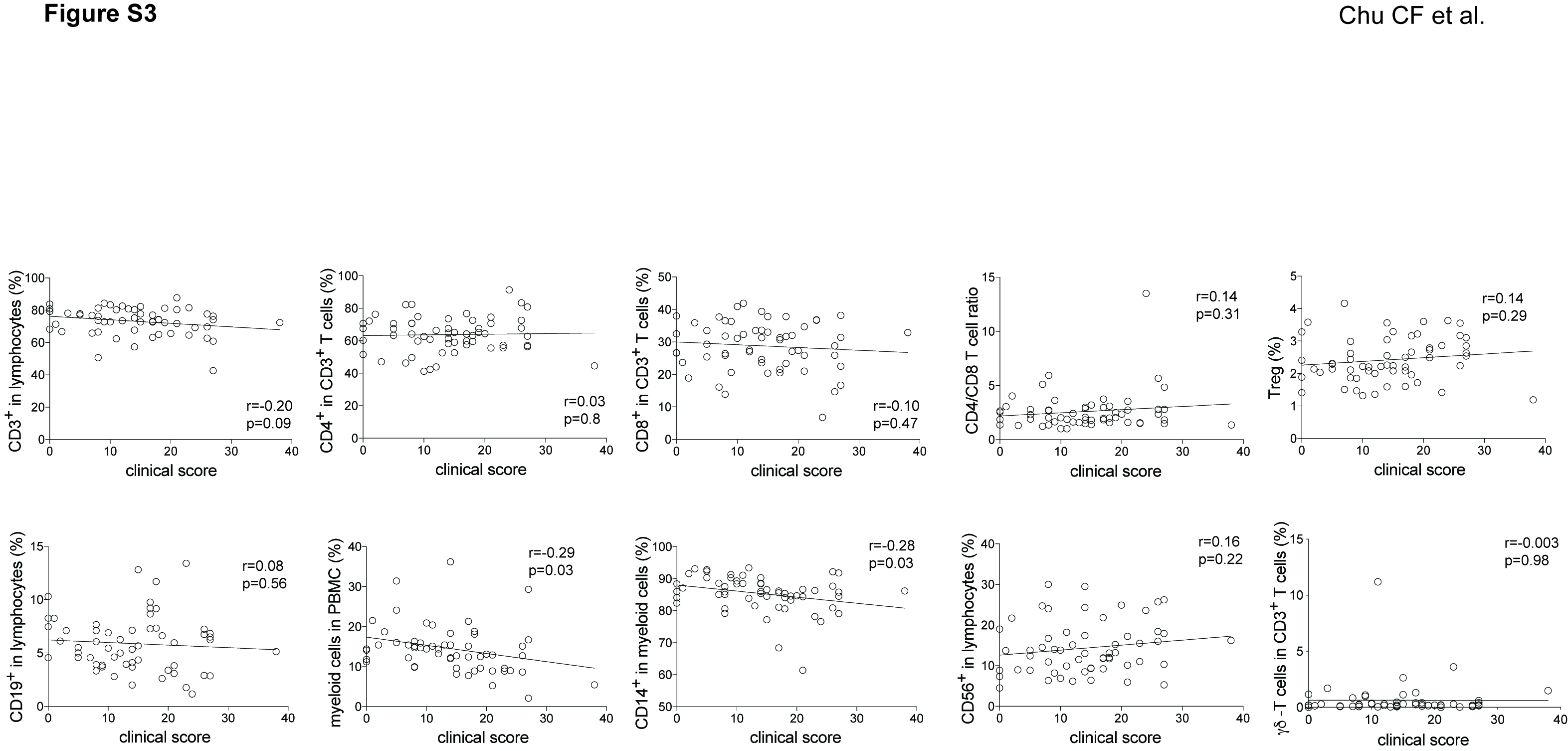

Supplement: Supplementary Figure 3 — Correlation of immunophenotypes with COVID-19 disease severity scores. Flow cytometry of ex vivo isolated PBMC gated on the indicated subpopulations. Each circle represents one patient. r, Pearson correlation coefficient, p, p-value. [file Image_3.tif]

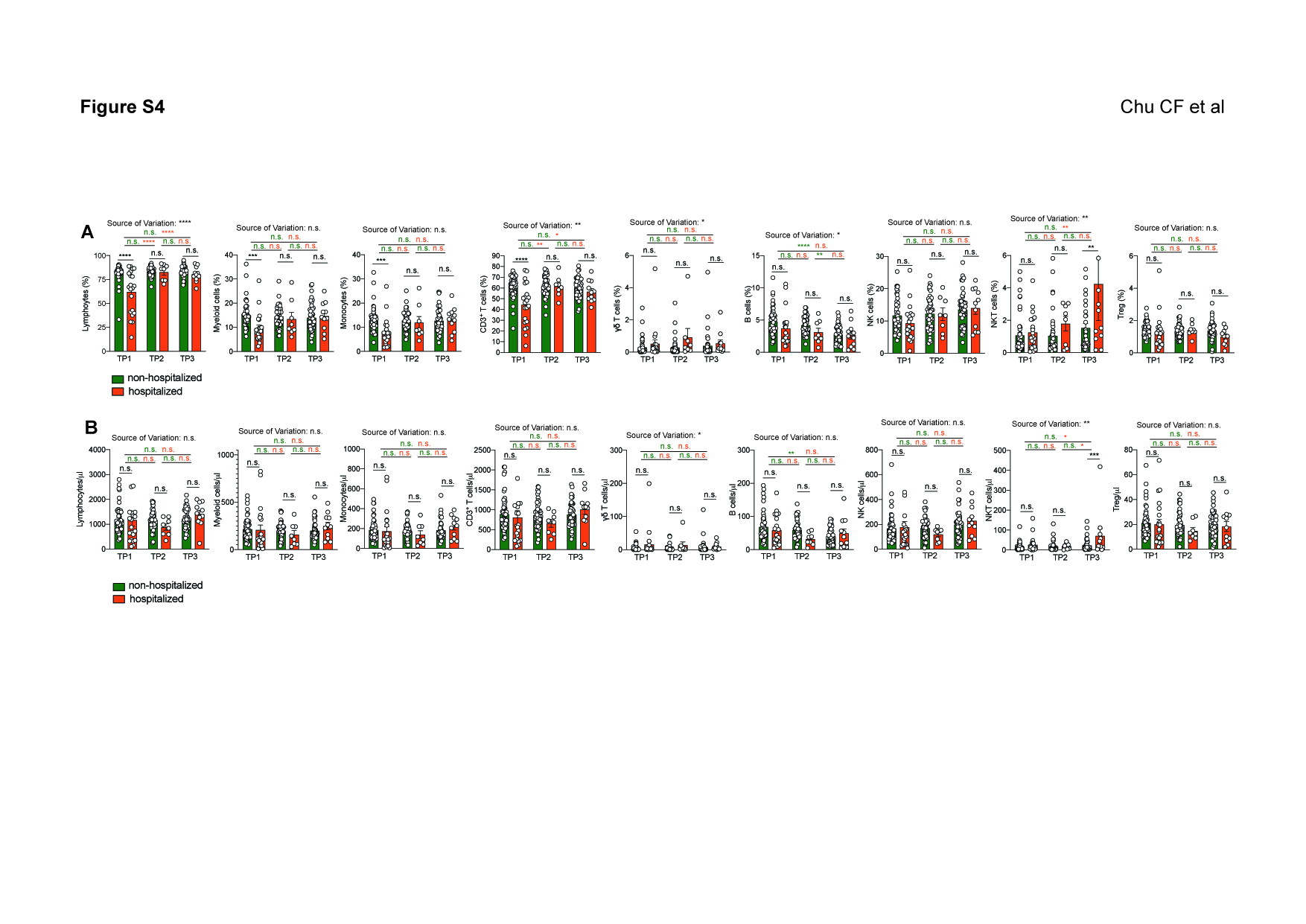

Supplement: Supplementary Figure 4 — Correlation of immunophenotypes with the hospitalization state over time. (A, B) Flow cytometry of ex vivo isolated PBMC gated on the indicated cellular subpopulations. Each circle represents one patient. (A) relative proportions as determined by manual gating. (B) absolute cell numbers as determined by manual gating. 2way ANOVA tests with Holm-Sidak’s multiple comparisons tests were performed for comparison of mildly versus severely affected patients longitudinally. n.s., not significant. [file Image_4.tif]

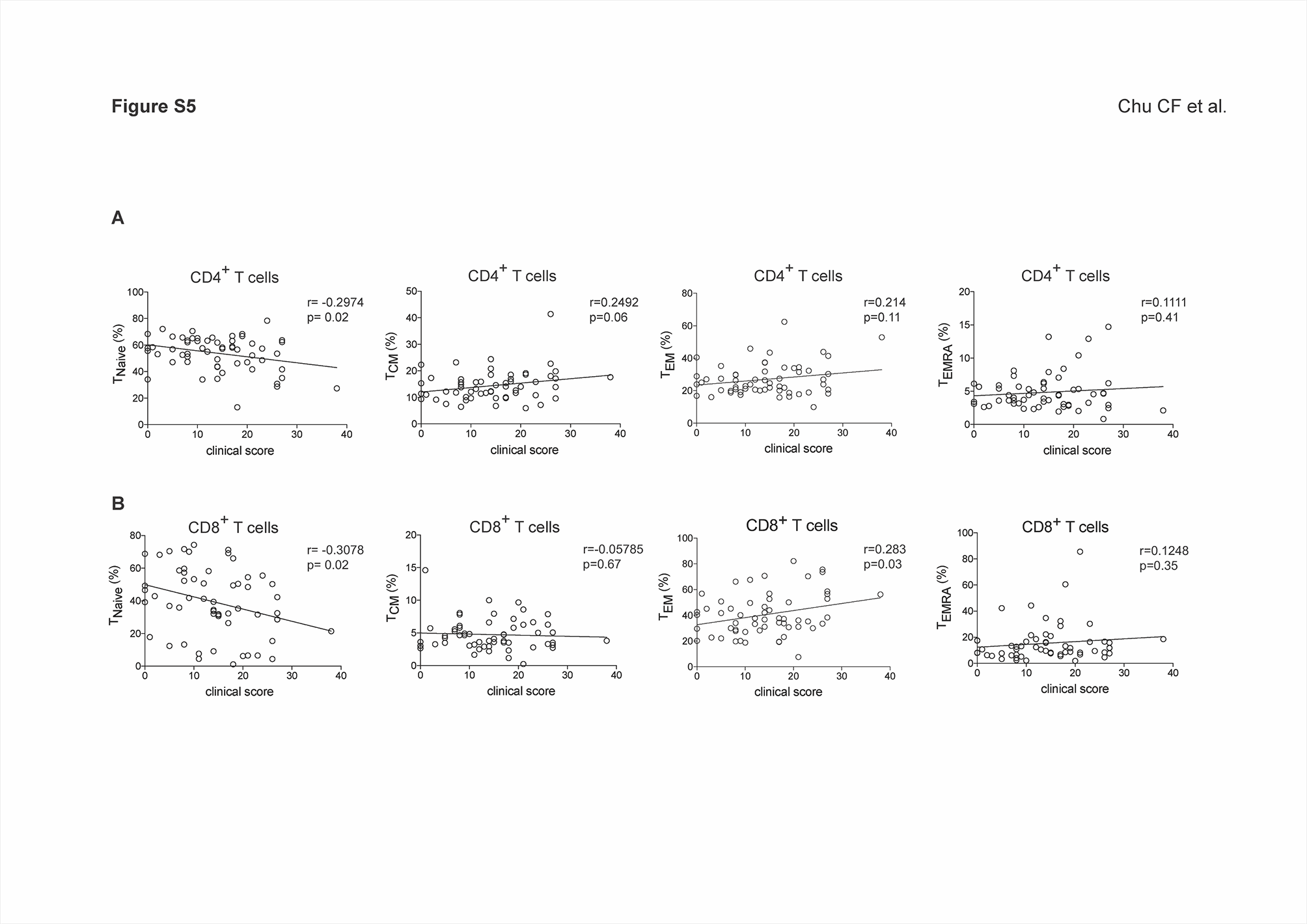

Supplement: Supplementary Figure 5 — Correlation of T cell differentiation states with COVID-19 disease severity scores. Flow cytometry of ex vivo isolated PBMC gated on the indicated subpopulations. Each circle represents one patient. r, Pearson correlation coefficient, p, p-value. [file Image_5.tif]

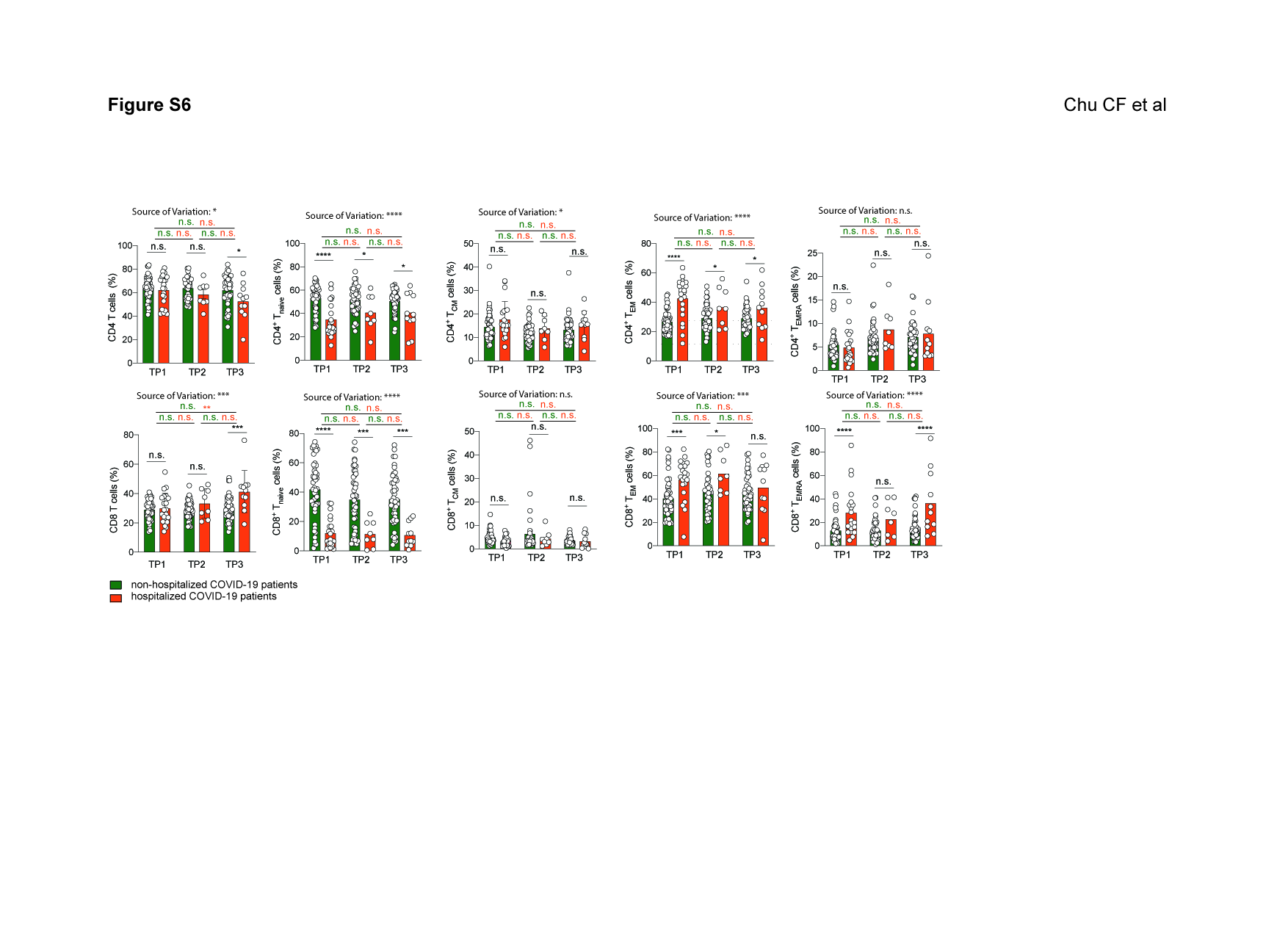

Supplement: Supplementary Figure 6 — Correlation COVID-19 hospitalization and disease severity with T cell differentiation states. (A, B) Flow cytometry of ex vivo isolated PBMC gated on CD3+CD19–CD56–γδ T cell– CD8+ or CD4+ T cells in hospitalized versus non-hospitalized patients. TNaive: CCR7+CD45RA+, TCM: CCR7+CD45RA, TEM: CCR7–CD45RA–, TEMRA: CCR7–CD45RA+. Each circle indicates one patient. Shown are relative proportions as determined by manual gating. 2way ANOVA tests with Holm-Sidak’s multiple comparisons tests were performed for comparison of mildly versus severely affected patients longitudinally. n.s., not significant. [file Image_6.tif]

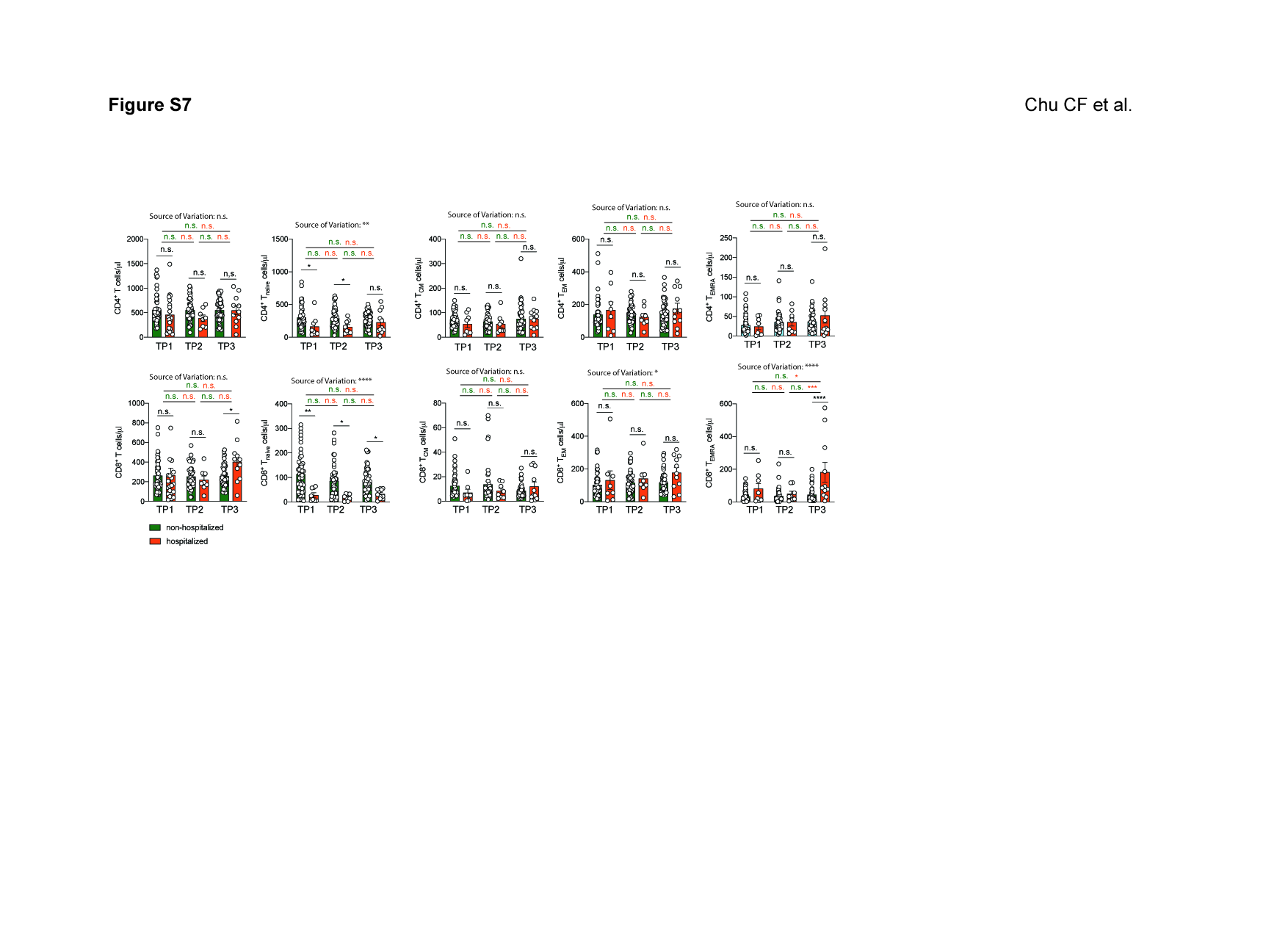

Supplement: Supplementary Figure 7 — Analysis of absolute numbers of T cell subsets in the blood in COVID-19 patients stratified into hospitalization and disease severity. (A, B) The analysis was performed as in S7. Shown are absolute cell numbers per μl blood. A two-tailed unpaired t test was performed for comparison of the respective patient groups. n.s., not significant. [file Image_7.tif]

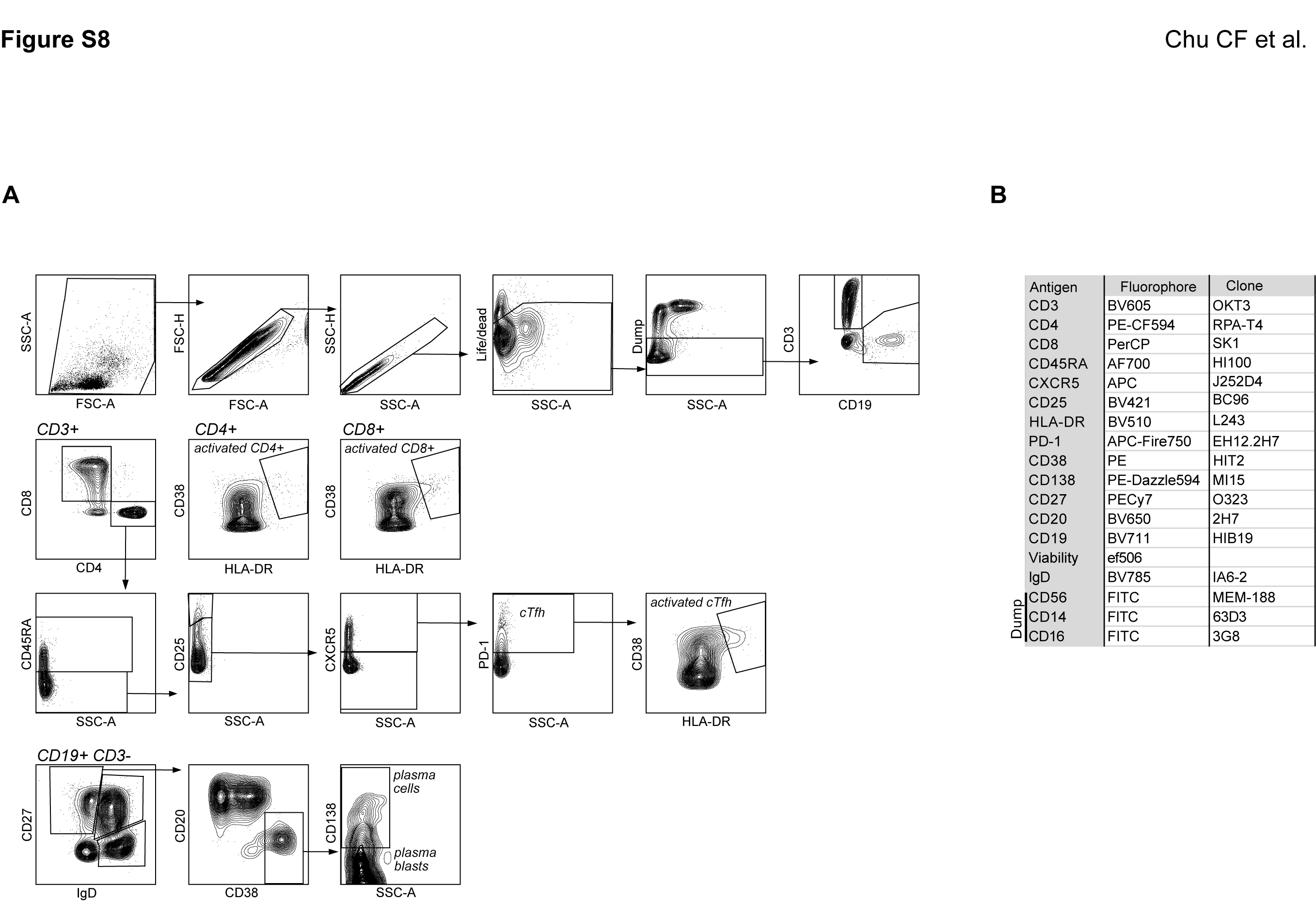

Supplement: Supplementary Figure 8 — Gating strategy for cellular markers of acute infection. (A) Shown is a representative gating strategy by spectral flow cytometry for one patient (left panel). (B) antibodies and their respective fluorochromes (right panel). [file Image_8.tif]

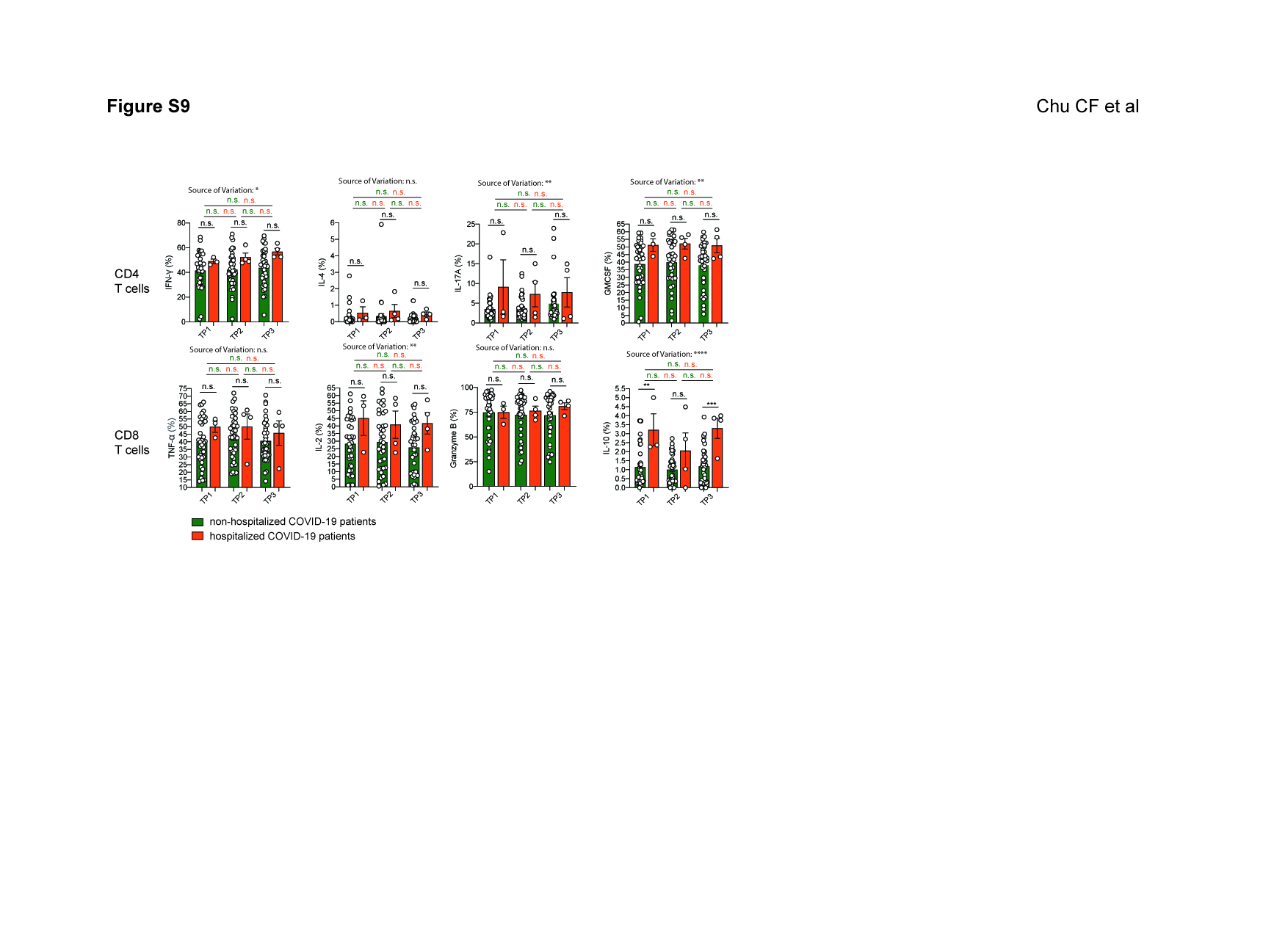

Supplement: Supplementary Figure 9 — Analysis of cytokine production in COVID-19 patients stratified into hospitalization and disease severity. Intracellular cytokine staining and flow cytometry of PBMC gated on CD3+ and CD4+ versus CD8+ T cells after 72 h of stimulation with CD3 and CD28 mAbs in hospitalized versus non-hospitalized COVID-19 patients. Each circle represents one patient. 2way ANOVA tests with Holm-Sidak’s multiple comparisons tests were performed for comparison of mildly versus severely affected patients longitudinally. n.s., not significant. [file Image_9.tif]

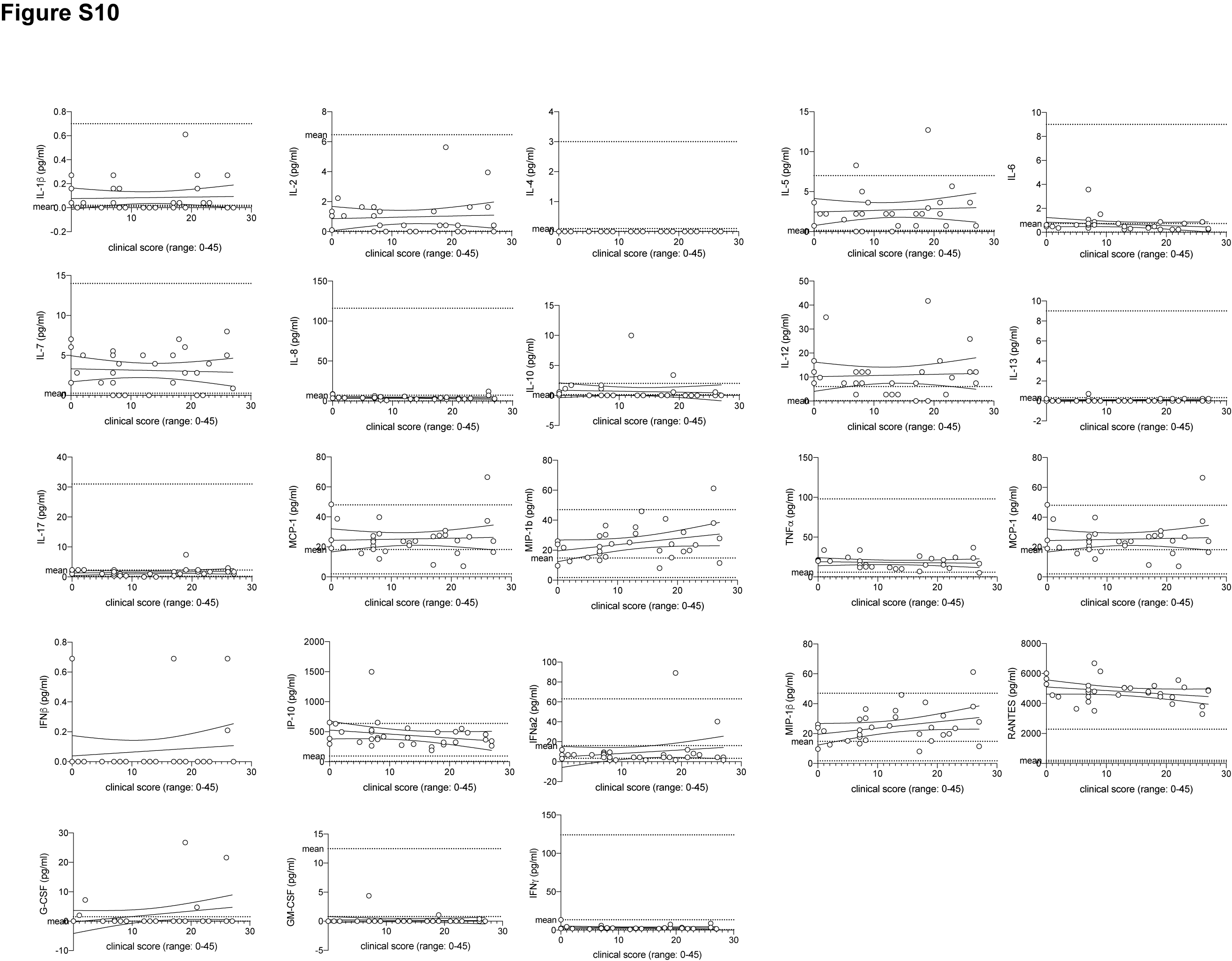

Supplement: Supplementary Figure 10 — Correlation of serum cytokines with COVID-19 disease severity. Serum was analyzed for cytokines using the multiplex bead array system Bio-Plex. Each circle represents one patient. Horizontal dotted lines indicate the physiological range of cytokine concentrations (low and high range and mean). r, Pearson correlation coefficient, p, p-value. [file Image_10.tif]

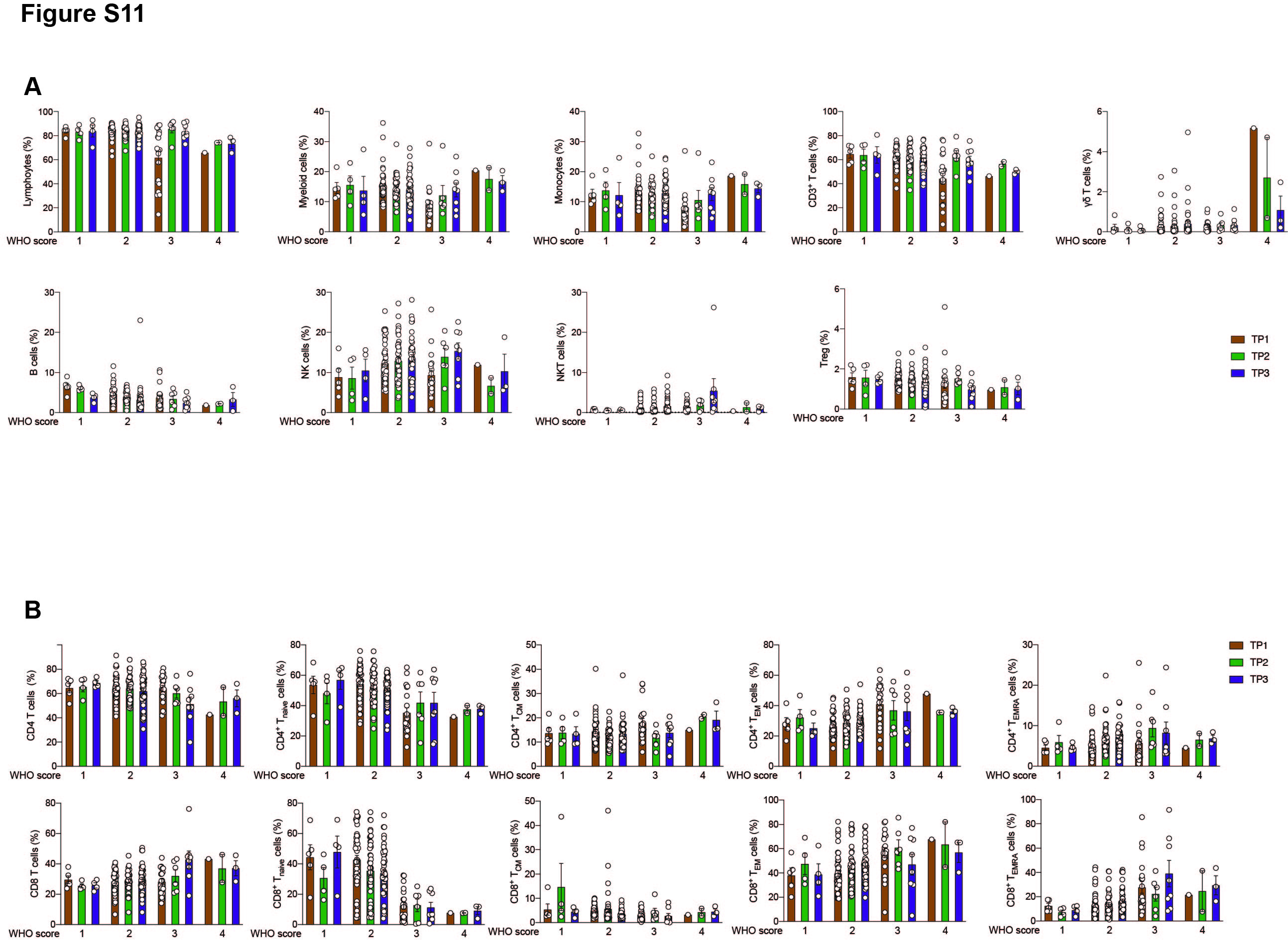

Supplement: Supplementary Figure 11 — Analysis of the relative proportions of immune cell subsets and T cell differentiation states in convalescent COVID-19 patients stratified according to the WHO Ordinal Scale for Clinical Improvement. Flow cytometry of ex vivo isolated PBMC gated on the indicated cellular subpopulations. Each circle represents one patient. Shown are the relative proportions of immune cell subsets. (A) Shown are immune cell types. (B) Shown are T cell differentiation states. [file Image_11.jpg]

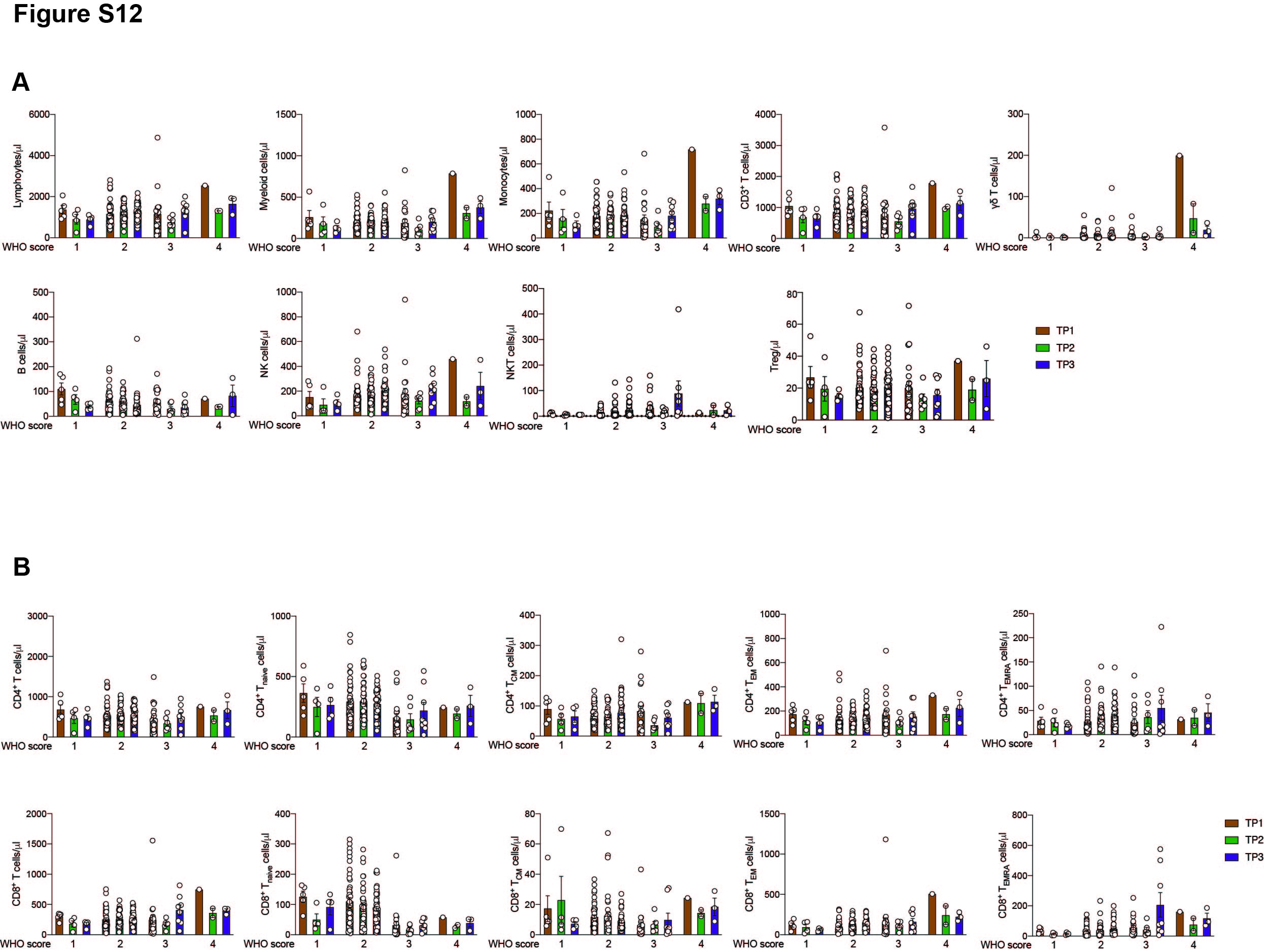

Supplement: Supplementary Figure 12 — Analysis of the relative proportions of absolute numbers of immune cell subsets and T cells in distinct differentiation states in convalescent COVID-19 patients stratified according to the WHO Ordinal Scale for Clinical Improvement. Flow cytometry of ex vivo isolated PBMC gated on the indicated cellular subpopulations. Each circle represents one patient. Shown are the absolute cell numbers of immune cell subsets. (A) Shown are immune cell types. (B) Shown are T cell differentiation states. [file Image_12.jpg]
