# Supplementary material for: Convalescent COVID-19 Patients Without Comorbidities Display Similar Immunophenotypes Over Time Despite Divergent Disease Severities
Source: Front Immunol. 2021 Aug 19;12:601080. doi: 10.3389/fimmu.2021.601080 (PMC8634761; doi:10.3389/fimmu.2021.601080)
Supplement: Supplementary Table 1 — Patient information. [file Table_1.docx]

Table 1

| Patient number | Clinical score | WHO score | Age (years) | Sex | Hospitalisation |
| --- | --- | --- | --- | --- | --- |
| 1 | 0 | 1 | 29 | male | no |
| 2 | 0 | 1 | 23 | female | no |
| 3 | 18 | 2 | 33 | male | no |
| 4 | 1 | 1 | 56 | female | no |
| 5 | 21 | 2 | 24 | female | no |
| 6 | 8 | 2 | 24 | female | no |
| 7 | 17 | 2 | 25 | female | no |
| 8 | - | 3 | 78 | male | yes |
| 9 | 27 | 3 | 47 | male | yes |
| 10 | 18 | 3 | 72 | female | yes |
| 11 | 27 | 3 | 52 | male | yes |
| 13 | 26 | 2 | 59 | male | no |
| 14 | 12 | 2 | 22 | female | no |
| 15 | 8 | 2 | 59 | female | no |
| 16 | 17 | 3 | 42 | male | yes |
| 17 | 23 | 3 | 57 | male | yes |
| 19 | - | 3 | 75 | male | yes |
| 21 | 0 | 1 | 24 | female | no |
| 23 | - | 3 | 83 | male | yes |
| 24 | 17 | 2 | 23 | male | no |
| 25 | 11 | 2 | 54 | male | no |
| 26 | 18 | 2 | 48 | male | no |
| 27 | - | 3 | 60 | male | yes |
| 28 | - | 3 | 80 | female | yes |
| 29 | 7 | 2 | 22 | male | no |
| 30 | 21 | 2 | 36 | female | no |
| 31 | 14 | 2 | 37 | male | no |
| 32 | 8 | 2 | 25 | male | no |
| 33 | 14 | 2 | 43 | female | no |
| 34 | 0 | 1 | 28 | female | no |
| 35 | 19 | 2 | 34 | male | no |
| 36 | 13 | 2 | 25 | male | no |
| 37 | 21 | 3 | 55 | male | yes |
| 38 | 7 | 2 | 60 | male | no |
| 39 | 7 | 2 | 22 | male | no |
| 40 | 26 | 2 | 65 | male | no |
| 41 | 14 | 2 | 60 | female | no |
| 42 | 7 | 2 | 54 | female | no |
| 43 | 5 | 2 | 25 | male | no |
| 44 | 23 | 2 | 37 | female | no |
| 45 | 8 | 2 | 65 | female | no |
| 46 | 19 | 2 | 31 | male | no |
| 47 | 26 | 2 | 46 | female | no |
| 48 | 27 | 2 | 42 | female | no |
| 49 | 14 | 2 | 54 | male | no |
| 50 | 9 | 2 | 33 | female | no |
| 51 | 2 | 2 | 25 | female | no |
| 52 | 15 | 2 | 25 | female | no |
| 54 | 13 | 2 | 51 | male | no |
| 55 | 10 | 2 | 21 | female | no |
| 60 | - | 3 | 51 | male | yes |
| 62 | - | 3 | 80 | female | yes |
| 63 | - | 3 | 64 | male | yes |
| 64 | 14 | 2 | 65 | female | no |
| 65 | 15 | 2 | 53 | female | no |
| 66 | 7 | 2 | 53 | male | no |
| 67 | 11 | 4 | 65 | male | yes |
| 68 | 22 | 2 | 44 | female | no |
| 69 | 15 | 2 | 52 | female | no |
| 70 | 27 | 2 | 43 | female | no |
| 72 | 15 | 2 | 42 | male | no |
| 75 | 38 | 3 | 41 | female | yes |
| 76 | - | 3 | 79 | female | yes |
| 77 | 3 | 2 | 21 | male | no |
| 82 | 10 | 2 | 19 | female | no |
| 89 | 20 | 2 | 43 | male | no |
| 113 | - | 3 | 56 | male | yes |
| 124 | 23 | 2 | 80 | female | no |
| 127 | - | 3 | 39 | male | yes |
| 138 | 14 | 3 | 34 | male | yes |
| 176 | 18 | 4 | 55 | female | yes |
| 183 | - | 3 | 59 | male | yes |
| 184 | - | 3 | 68 | female | yes |
| 189 | 23 | 4 | 46 | male | yes |
